# Heavy-Atom Effect Modulated Photoluminescence Properties of Trinuclear Copper(I) Clusters with Haloarylacetylene Ligands

**DOI:** 10.3390/molecules31060987

**Published:** 2026-03-15

**Authors:** Qiao Yao, Ling-Ling Cai, Fang-Xue Xiao, Yu-Ting Ma, Shi-Yang Li, Jun Yi, Yao Wang

**Affiliations:** 1 College of Chemistry and Materials Science, Fujian Normal University, Fuzhou 350108, China; yaoqiao@fjirsm.ac.cn (Q.Y.);; 2 State Key Laboratory of Structural Chemistry, Fujian Institute of Research on the Structure of Matter, Chinese Academy of Sciences, Fuzhou 350002, China

**Keywords:** heavy-atom effect, copper(I) clusters, photoluminescence, structure–property relationship

## Abstract

Copper(I) clusters have attracted significant interest due to their ultrasmall size, excellent photostability, large Stokes shift, and long emission lifetime. However, research on their structure–property relationship remains limited. In this study, we synthesized and comprehensively characterized a series of trinuclear copper(I) clusters, [Cu_3_(dppm)_3_(C≡CC_6_H_4_X)_2_]PF_6_ [Cu_3_-X, X = F, Cl, Br, and I, dppm = bis(diphenylphosphino)methane], in order to investigate the effect of ligands containing heavy atoms on photoluminescence. All clusters have the same triangular metal core and similar distribution of ligands, with the only difference being the substituent on the phenyl ring of the arylacetylene ligand. Owing to the heavy-atom effect, a notable Stokes shift was observed in the emission spectra of the clusters. Specifically, the emission peak of **Cu_3_-I** reached 564 nm, representing a 73 nm red shift compared to that of **Cu_3_-F**. Furthermore, **Cu_3_-Br** showed an absolute photoluminescence quantum yield of 15.24% and a lifetime of 114.56 μs, corresponding to 3.3-fold and 3.7-fold increases over the values for **Cu_3_-F**. This study provides novel insights into the heavy-atom effect of surface ligands on the luminescence of copper(I) clusters and offers a robust platform for probing their structure–property relationships.

## 1. Introduction

Nanotechnology advances are driven by precision synthetic method development, in-depth characterization of sophisticated nanomaterials, and targeted exploration of their structure–property relationships [1,2,3]. Ligand-protected metal clusters, a prototypical class of nanomaterials, have garnered significant interest in view of their precisely controllable synthesis and tunable properties [4,5,6,7]. Among them, coinage metal (gold, silver and copper) clusters exhibit unique properties according to composition of metal core as well as the specific characteristics of ligands, including advanced optics, magnetic properties, biomedicine, and catalytic activity [8,9,10,11,12,13,14,15,16,17,18]. Copper(I) clusters have attracted significant interest in the field of materials science due to ultrasmall size, excellent photostability, large Stokes shift, long emission lifetime, and outstanding biocompatibility [19,20,21].

The heavy-atom effect (HAE) in metal clusters is a critical phenomenon influencing their photophysical properties, particularly phosphorescence and intersystem crossing (ISC), due to enhanced spin-orbit coupling (SOC) [22,23,24]. The presence of heavy atoms promotes the transition from the singlet excited state (S_1_) to the triplet excited state (T_1_), thus increasing the efficiency of phosphorescence and significantly affecting both photoluminescence quantum yield and lifetime [25]. Recent research on silver nanoclusters embedded in amorphous matrices demonstrated that the incorporation of halogens (Cl, Br, I) significantly enhanced phosphorescence, achieving a quantum yield of 27.54% through strengthened SOC and accelerated ISC [26].

However, the HAE is not always straightforward. In some cases, an anti-heavy-atom effect has been observed, where the introduction of heavier halogens leads to a decrease in the photoluminescence quantum yield [27]. For instance, bimetallic nanoclusters of the type Au_28_Cu_12_X_4_Cl_4_ (X = Cl, Br, and I) exhibited strong near-infrared emission, but the introduction of heavier halogens (Br, I) resulted in a decrease in photoluminescence quantum yield (PLQY), contrary to the expected enhancement from HAE [28]. The primary cause of this phenomenon may be that the larger atomic sizes of heavier halides induce expansion of the cluster core, resulting in an increased non-radiative transition rate. This suggests a complex interplay between electronic structure, SOC, and other deactivation pathways, such as non-radiative decay. While HAE can significantly enhance ISC efficiency and triplet exciton population, it can also accelerate radiative decay, potentially reducing phosphorescence lifetime. Moreover, tunable surface ligands offer a powerful means to design clusters with tailored properties [29,30]. While introducing heavy atoms onto ligands is known to influence cluster luminescence, systematic studies remain scarce [31,32]. Thus, to establish clear structure–property relationships, there is a compelling need for well-defined model clusters.

Herein, we developed a series of trinuclear copper(I) complexes, [Cu_3_(dppm)_3_(C≡CC_6_H_4_X)_2_]PF_6_ [Cu_3_-X, X = F, Cl, Br, and I, dppm = bis(diphenylphosphino)-methane], designed as a model system to examine the effect of ligands containing heavy atoms on the photoluminescence properties of copper(I) clusters [33,34]. These clusters possess an identical trigonal-planar Cu_3_ core and coordination geometry, differing only in the substituent groups on the arylacetylene ligands. Notably, these heavy atom substituents significantly influence the band structure and the luminescence properties of the clusters by modulating the SOC strength and the nature of excited-state transitions. Theoretical and experimental results confirm that the luminescence of Cu_3_-X clusters originates from metal-to-ligand charge transfer (MLCT) mixed with partial ligand-to-ligand charge transfer (LLCT) triplet excited states, with halogen p-orbitals participating in the composition of the conduction band and regulating the energy level of the excited state. Specifically, with the introduction of heavier atoms, the emission peak progressively red-shifted from 491 nm (**Cu_3_-F**) to 564 nm (**Cu_3_-I**), corresponding to a red shift of 73 nm. In addition, the absolute PLQY and lifetime of **Cu_3_-Br** were determined to be 15.24% and 114.56 μs, respectively, marking a 3.3-fold enhancement in PLQY and a 3.7-fold extension in lifetime relative to **Cu_3_-F**. These observations highlight the significant impact of heavy atoms on the phosphorescent properties of the copper clusters by tuning the ISC efficiency and non-radiative decay pathways. The study underscores the role of the HAE of surface ligands in modulating the luminescence behavior of clusters and providing an excellent model platform for investigating structure–property relationships in cluster systems.

## 2. Results and Discussion

### 2.1. Crystal Structure of Cu_3_-X

**Figure 1:**
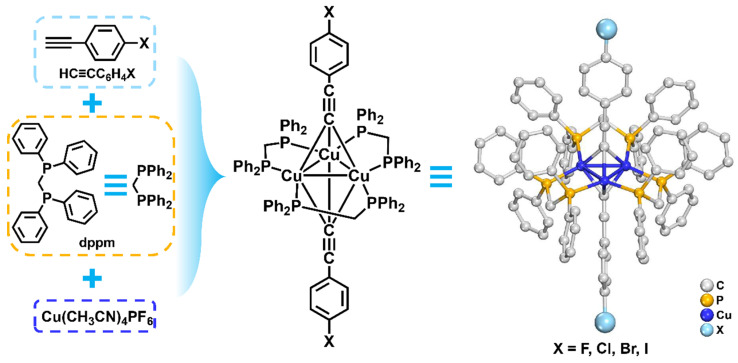
Scheme of synthetic rotes of Cu_3_-X, X = F, Cl, Br, and I.

All trinuclear copper(I) clusters were synthesized utilizing a one-pot synthesis approach. Take **Cu_3_-F** as an example, Cu(CH_3_CN)_4_PF_6_, 4-Fluorophenylacetylene, and dppm were dissolved in a solvent mixture comprising Et_3_N and DCM (Figure 1). The resultant mixture was agitated overnight at ambient temperature. Subsequently, this solution underwent centrifugation and diffusion with ether, culminating in the production of white crystals as the final products. **Cu_3_-Cl**, **Cu_3_-Br**, and **Cu_3_-I** can be obtained using an analogous procedure by replacing 4-fluorophenylacetylene with the corresponding haloarylacetylene.

**Figure 2:**
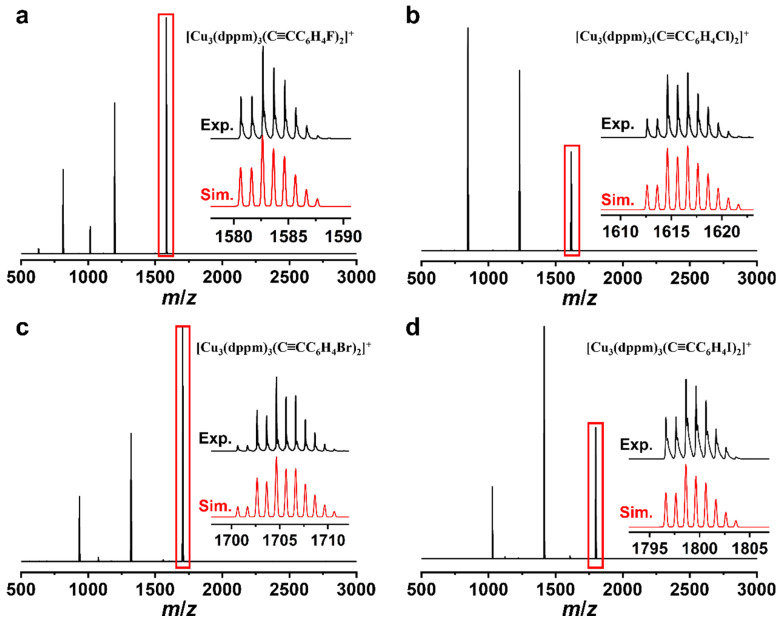
HR-ESI-MS spectra of (**a**) **Cu_3_-F**, (**b**) **Cu_3_-Cl**, (**c**) **Cu_3_-Br**, and (**d**) **Cu_3_-I** with insets showing a comparison between the experimental (Exp.) and simulated (Sim.) isotope patterns for the component signals in the red boxes.

The ^1^H and ^31^P{^1^H} NMR spectroscopic results evidenced the successful formation of trinuclear copper(I) clusters (Figures S1–S12). A systematic downfield shift of the ^1^H nuclei adjacent to the halogen atoms is observed, and the magnitude of this shift increases progressively from F to Cl, Br, and I, which is attributed to the enhanced Spin-Orbit Heavy Atom on the Light Atom effect with increasing halogen atomic weight [35]. ^1^H DOSY spectroscopy revealed that all protons of the clusters possessed identical diffusion coefficients (Figures S13–S16). The high-resolution electrospray-ionization mass spectrometry (HR-ESI-MS) analyses further confirmed the chemical formula of Cu_3_-X clusters. The spectrum of Cu_3_-X features a series of peaks corresponding to +1 charged species with progressive loss of counter anions and dppm ligands. As shown in Figure 2, the selected molecular ion peaks at *m*/*z* 1582.6639, 1616.8998, 1704.6508, and 1798.6416 of **Cu_3_-F**, **Cu_3_-Cl**, **Cu_3_-Br**, and **Cu_3_-I**, respectively, which corresponding to the [Cu_3_(dppm)_3_(C≡CC_6_H_4_F)_2_]^+^ (calcd. *m*/*z* 1582.2151), [Cu_3_(dppm)_3_(C≡CC_6_H_4_Cl)_2_]^+^ (calcd. *m*/*z* 1616.1559), [Cu_3_(dppm)_3_(C≡CC_6_H_4_Br)_2_]^+^ (calcd. *m*/*z* 1704.0549), and [Cu_3_(dppm)_3_(C≡CC_6_H_4_I)_2_]^+^ (calcd. *m*/*z* 1798.0272). Additional ion peaks observed in the HR-ESI-MS spectra and their corresponding species are summarized in Figures S17–S20. Notably, the HR-ESI-MS spectrum of **Cu_3_-F** shows one peak at *m*/*z* 1016.0740 corresponding to [Cu_2_(dppm)_2_(C≡CC_6_H_4_F)]^+^ (calcd. *m*/*z* 1016.1360) with the loss of 1 C≡CC_6_H_4_F, 1 dppm, 1 Cu and counter anion (Figure S17c).

**Figure 3:**
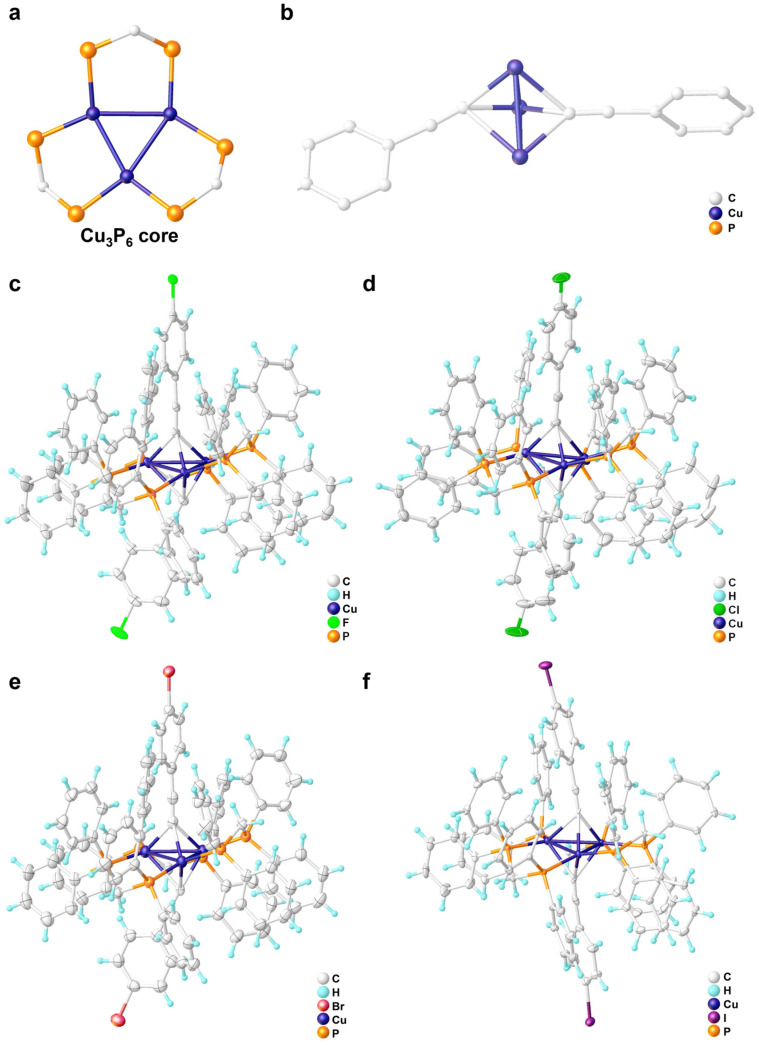
(**a**) The trigonal-planar Cu_3_ core and Cu_3_P_6_ core structure. (**b**) The *μ*_3_-*η*^1^ bridging mode of haloarylacetylene ligands, hydrogen atoms are omitted for clarity. Molecular structure of (**c**) **Cu_3_-F**, (**d**) **Cu_3_-Cl**, (**e**) **Cu_3_-Br**, and (**f**) **Cu_3_-I**.

The structures of the crystals were further determined by single-crystal X-ray analysis. **Cu_3_-Cl** and **Cu_3_-Br** were found to crystallize in the triclinic P-1 space group, while **Cu_3_-F** and **Cu_3_-I** crystallized in the monoclinic Cc space group and the monoclinic P2_1_/n space group, respectively. Crystallographic data and selected bond lengths and angles are listed in Tables S1–S12. All clusters exhibit an identical structural framework. As shown in Figure 3a, all trinuclear copper(I) complexes feature a trigonal-planar Cu_3_ core, with Cu–Cu distances ranging from 2.51 to 2.72 Å. This value is shorter than the van der Waals radius sum of copper atoms (2.8 Å), thus confirming the existence of metal–metal interactions. Furthermore, each dppm ligand bridges two copper atoms with an average Cu–P bond distances of 2.28 Å, whereby three ligands collectively form the Cu_3_P_6_ core structure. Two alkynyl ligands were each σ-bonded to three copper atoms in the *μ*_3_-*η*^1^ bridging modes, with Cu–C distances ranging from 2.07 to 2.46 Å (Figure 3b). Notably, the axes of the alkyne triple bonds were not perfectly perpendicular to the Cu_3_ plane, but rather tilted slightly toward the Cu–C edges with the longest Cu–C bond lengths. Specifically, the angles of the C≡C bond relative to the Cu_3_ plane were measured to be (84.79°, 74.65°) for **Cu_3_-F**, (90.00°, 62.25°) for **Cu_3_-Cl**, (90.00°, 61.44°) for **Cu_3_-Br**, and (78.30°, 80.54°) for **Cu_3_-I**.

### 2.2. Photophysical Property and Theoretical Calculations

**Figure 4:**
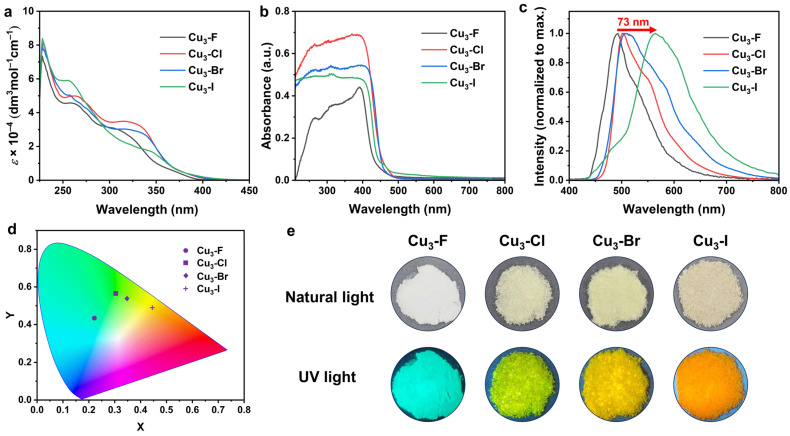
(**a**) UV-Vis absorption spectra of Cu_3_-X (conc. 1 × 10^−5^ mol L^−1^) in CH_2_Cl_2_. (**b**) Solid-state UV-Vis absorption spectra of Cu_3_-X. (**c**) Photoluminescence spectra of Cu_3_-X powders. (**d**) CIE coordinates of **Cu_3_-F** (circle), **Cu_3_-Cl** (square), **Cu_3_-Br** (rhombus) and **Cu_3_-I** (plus). (**e**) Images of the Cu_3_-X powders coated under natural light and 365 nm UV light.

The electronic absorption spectra of the trinuclear copper(I) complexes in CH_2_Cl_2_ exhibit absorption bands at approximately 230–250 nm and 250–320 nm (Figure 4a). The high-energy absorptions around 230–250 nm occur at positions similar to the absorption maxima of the free dppm ligand and are assigned to intraligand transitions (Figure S21). The lower-energy absorption in the range of about 250–310 nm is tentatively attributed to acetylide ligand-centered absorption, given its similarity in energy to those of free haloarylacetylene (Figure S21). Additionally, low-energy absorptions are observed in the region of approximately 320–400 nm. It is noteworthy that due to the HAE, **Cu_3_-F** exhibits a higher-energy absorption at approximately 316 nm, while with the substitution of heavier atoms, **Cu_3_-Cl**, **Cu_3_-Br**, and **Cu_3_-I** display progressively lower-energy absorptions at 326 nm, 332 nm, and 346 nm, respectively. In addition, the UV-Vis diffuse reflectance spectra of Cu_3_-X were recorded in the range of 200–800 nm at room temperature (Figure S22). For a better visualization of the absorption features, the reflectance data were converted to −lg(R) values, which serve as a qualitative representation of the absorbance for solid powder samples. All Cu_3_-X clusters exhibit strong absorption in the 200–450 nm range, with negligible visible-light absorption above 450 nm (Figure 4b). The similar shape of all absorption curves suggests consistent electronic transition behaviors, dominated by charge-transfer transitions from the valence band to the conduction band. The data were subsequently transformed via the Kubelka–Munk function, from which the optical band gap energies were derived (Figure S23). The optical band gaps of **Cu_3_-F**, **Cu_3_-Cl**, **Cu_3_-Br**, and **Cu_3_-I** are 2.94 eV, 2.83 eV, 2.79 eV and 2.84 eV, respectively. These results clearly demonstrate that halogen substitution effectively modulates the optical absorption properties of the copper-based clusters.

First-principles calculations based on density functional theory (DFT) were carried out for Cu_3_ clusters using the Vienna Ab initio Simulation Package (VASP). The electronic states at the valence band maximum (VBM) are predominantly derived from the Cu 3d and C 2p orbitals, whereas the conduction band minimum (CBM) is primarily composed of states from organic ligands, dominated by C 2p orbitals. Given the incorporation of halide atoms (F, Cl, Br, I) within the organic ligands, the contributions of halide p-orbitals (F 2p, Cl 3p, Br 4p, and I 5p) were additionally evaluated in the calculations. As illustrated in Figure S24, the heavier halogen atoms (Cl, Br and I) contribute to the conduction band. Based on these electronic structure characteristics, the emission mechanisms of the four complexes are assigned to either LLCT or MLCT processes.

The photoluminescence (PL) characteristics of the trinuclear copper(I) complexes were subsequently investigated. The photoluminescence spectra of solid of Cu_3_-X at room temperature are shown in Figure 4c, and **Cu_3_-F** shows a strong blue emission at 491 nm with an excitation wavelength of 320 nm. Notably, clusters built from ligands with heavier substituents display a pronounced redshift, as their emission maxima are shifted to considerably longer wavelengths. Specifically, **Cu_3_-Cl**, **Cu_3_-Br**, and **Cu_3_-I** showed a single-band emission peak at 502 nm, 511 nm, and 564 nm at excitation wavelengths of 320 nm, respectively. The Commission Internationale de L’Eclairage color coordinates (CIE) of **Cu_3_-F**, **Cu_3_-Cl**, **Cu_3_-Br**, and **Cu_3_-I** are (0.22, 0.43), (0.30, 0.56), (0.35, 0.53), and (0.44, 0.49), respectively (Figure 4d). As shown in Figure 4e, the luminescence color of Cu_3_-X clusters can be better adjusted by the controlled modification of organic ligands.

To evaluate the luminescence properties of Cu_3_-X clusters, the absolute PLQY were measured at room temperature. The PLQY of **Cu_3_-F**, **Cu_3_-Cl**, **Cu_3_-Br**, and **Cu_3_-I** were 4.61%, 2.97%, 15.24% and 0.19%, respectively. At the excitation wavelengths of Cu_3_-X clusters, the luminescence lifetime decay curves of the complexes were tested in their maximum emission wavelengths and emission ranges, and exponential fitting was performed (Figure S25). The luminescence lifetimes of **Cu_3_-F**, **Cu_3_-Cl**, **Cu_3_-Br**, and **Cu_3_-I** were calculated to be 30.64 μs, 72.19 μs, 114.56 μs and 18.08 μs, respectively. The microsecond radiative decay lifetime confirms the phosphorescent character of the emission, which arises from the triplet excited states. For **Cu_3_-F**, **Cu_3_-Cl**, and **Cu_3_-Br,** the HAE strengthens progressively with the increasing atomic number of the halogen, leading to a corresponding increase in the PLQY and luminescence lifetime. Specifically, **Cu_3_-Br** exhibited a 3.3-fold higher PLQY and a 3.7-fold longer photoluminescence lifetime than **Cu_3_-F**. These results demonstrate that introducing heavy atoms into the ligands is a viable strategy to enhance the luminescence performance of the clusters through the HAE, as the enhanced SOC promotes intersystem crossing. However, **Cu_3_-I** exhibited an unexpected and sharp decrease in both PLQY and luminescence lifetime, deviating from the trend observed in the other clusters. We monitored the changes in the ^1^H and ^31^P{^1^H} NMR spectra of **Cu_3_-I** upon light irradiation. After exposure to 365 nm UV light for 2 min, the ^1^H NMR signals became broadened, and additional peaks appeared in the ^31^P{^1^H} NMR spectrum (Figure S26), suggesting that the structure of **Cu_3_-I** might be disrupted. This poor photostability of **Cu_3_-I** is responsible for its compromised optical performance. Moreover, the characteristic peaks of **Cu_3_-I** in both the ^1^H and ^31^P{^1^H} NMR spectra gradually vanished with increasing irradiation time. A new resonance at δ = 25 in the ^31^P{^1^H} NMR spectrum indicated the oxidation of dppm ligands to bis(diphenylphosphanyl)methane oxide, which abolished its chelating ability toward the Cu_3_ core and led to disassembly of the trinuclear cluster framework. Comparison with other low-nuclearity copper clusters reported in the literature reveals that the Cu_3_-X series in this work exhibits a remarkably wide tunability of phosphorescence lifetimes (Table S13). Notably, the lifetime of **Cu_3_-Br** (114.56 μs) is significantly longer than most entries in the table, such as [Cu_3_(dppm)_3_(C≡CC_6_H_4_OMe-*p*)_2_]PF_6_ (63.8 μs) [34] and [Cu_3_(dppm)_3_ (C≡CC_6_H_4_-4-NHC(O)NHC_6_H_4_-4-CF_3_)_2_F]_∞_ (54.1 μs) [20]. In contrast, the lifetime of **Cu_3_-I** (18.08 μs) is comparable to that of clusters bearing heavy atoms, as exemplified by Cu_4_I_4_L_4_ (6.6 μs) [36] and [Cu_4_I_4_(4-dpda)_4_] (2.54 μs) [37].

Subsequently, the photoluminescence characteristics of Cu_3_-X clusters were evaluated at a temperature of 77 K (Figure S27). At this temperature, all four compounds exhibit very similar emission spectra, with peaks in the blue-green-yellow region (~480–580 nm). Specifically, **Cu_3_-F** exhibited emission at a wavelength of 480 nm, accompanied by a shoulder at 527 nm. **Cu_3_-Cl** showed emission at 511 nm with a shoulder at 560 nm. **Cu_3_-Br** emitted at 512 nm, with a corresponding shoulder at 561 nm. For **Cu_3_-I**, the emission maximum was observed at 486 nm, accompanied by two shoulders at 531 and 586 nm. This indicates that non-radiative processes are frozen at 77 K, and the emission is dominated by the intrinsic radiative decay from T_1_. Moreover, the phosphorescence lifetimes of Cu_3_-X clusters follow the order: **Cu_3_-F** (363.20 µs) > **Cu_3_-Cl** (313.70 µs) ≈ **Cu_3_-Br** (313.70 µs) > **Cu_3_-I** (265.84 µs), which decrease progressively with increasing atomic number of the halogen (Table 1 and Figure S28). This trend is a classic manifestation of the HAE, where stronger SOC in the heavier halogens partially relaxes the spin-forbidden triplet-singlet transition, thereby accelerating the radiative rate and shortening the observed lifetime.

**Figure 5:**
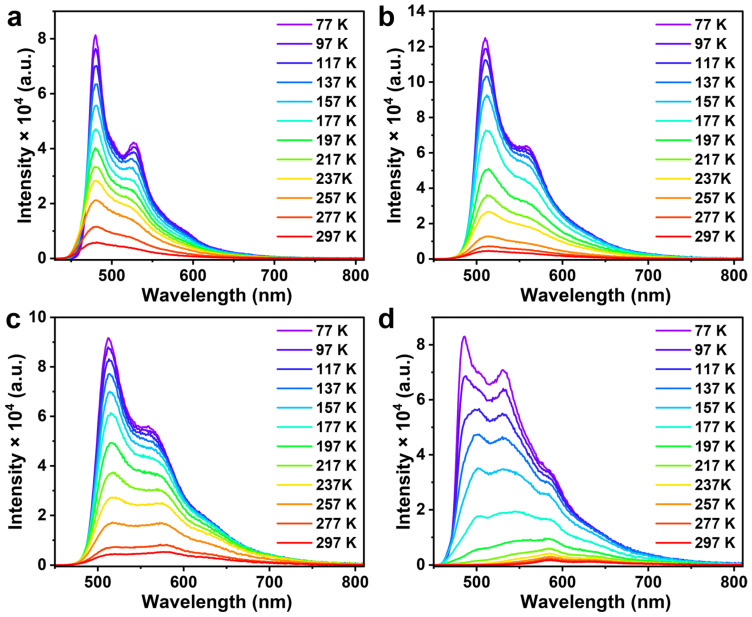
Temperature dependence of the emission spectra in the range of 77–297 K of (**a**) **Cu_3_-F**, (**b**) **Cu_3_-Cl**, (**c**) **Cu_3_-Br**, and (**d**) **Cu_3_-I**.

The temperature-dependent luminescence characteristics of the solid-state samples were assessed, revealing distinct luminescent behaviors for Cu_3_-X clusters at lower temperatures (Figure 5). As the temperature was raised from 77 K to 297 K, a significant decrease in both the intensity and lifetime of the phosphorescence was observed, which mainly attributed to the transition from radiative-dominated to non-radiative-dominated kinetics. For **Cu_3_-F** and **Cu_3_-Cl**, the pronounced rigidity of the crystal lattice and weak electron–phonon coupling effectively suppress excited-state structural relaxation. As a result, their room-temperature emission maxima remain nearly identical to those observed at 77 K, and their phosphorescence lifetimes are moderate. In contrast, **Cu_3_-Br** exhibits intermediate lattice flexibility and electron–phonon coupling strength, enabling partial relaxation of the excited state without leading to complete thermal quenching. Consequently, its emission peak undergoes a slight red-shift relative to the low-temperature position, yet it retains the longest room-temperature lifetime among Cu_3_-X clusters (114 µs), indicative of a relatively high activation energy barrier for non-radiative decay. **Cu_3_-I**, however, represents the extreme case within Cu_3_-X clusters. As the heaviest halogen, iodide introduces two synergistic effects that profoundly influence the photophysical behavior. First, the strong SOC inherent to iodine, while promoting phosphorescence at low temperatures, simultaneously exacerbates non-radiative recombination at elevated temperatures by enhancing spin–vibration coupling. Second, the large ionic radius and high polarizability of iodide ligands hinder dense crystal packing, resulting in an exceptionally soft lattice with reduced structural rigidity. This combination of strong spin–orbit coupling and loose packing permits extensive thermally induced structural reorganization of the excited state at ambient temperature, leading to the formation of a lower-energy self-trapped exciton (STE) state. This is manifested as a significantly red-shifted emission band that deviates entirely from the original low-temperature peak. Concurrently, such excessive structural relaxation facilitates highly efficient non-radiative dissipation pathways, yielding an extremely short lifetime (18 µs) and severely quenched luminescence at room temperature. In summary, systematic halogen substitution on the ligand represents an effective strategy to regulate the excited-state dynamics. Furthermore, the values of the radiative rate constant (*k*_r_) and non-radiative rate constant (*k*_nr_) were calculated to quantitatively assess the degree of non-radiative decay in the structures of Cu_3_-X (Table 1). Notably, **Cu_3_-I** exhibited the largest non-radiative rate constant (*k*_nr_ = 5.52 × 10^4^ s^−1^), whereas **Cu_3_-Br** displays the smallest *k*_nr_ value (7.4 × 10^3^ s^−1^) among the series. This pronounced difference further confirmed that the softer lattice and enhanced electron-phonon coupling imparted by the heavier iodide ligand dramatically accelerate non-radiative recombination, consistent with the severely quenched emission and ultrashort lifetime observed for **Cu_3_-I** at room temperature.

**Figure 6:**
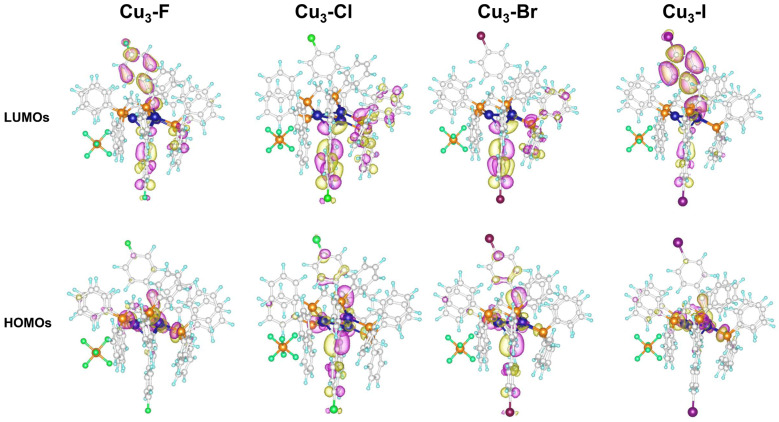
Key molecular orbitals contributing to the T_1_ excited state of Cu_3_-X Clusters.

To rationalize the experimental spectroscopic results at the molecular level, time-dependent density functional theory (TD-DFT) and spin–orbit coupling calculations were carried out. The T_1_ energies and SOC constants were calculated and listed in Table S14. The T_1_ excitation energies decrease gradually from **Cu_3_-F** to **Cu_3_-I**, consistent with the progressive red shift in emission wavelength. The SOC matrix elements between the lowest S_1_ and T_1_ were calculated to quantitatively evaluate the ISC process, which governs the phosphorescence emission. Notably, the SOC constant increases significantly from 1.59 cm^−1^ (**Cu_3_-F**) to 14.6 cm^−1^ (**Cu_3_-I**), providing direct quantitative evidence for HAE. The transition orbital analysis (Table S15) indicates that the T_1_ state possesses dominant MLCT character (Figure 6), with notable halogen contribution. These results clearly demonstrate that the halogen ligands modulate the emission energy and photophysical behavior via tuning the excited-state energy and SOC strength.

## 3. Materials and Methods

Unless otherwise stated, all reagents were purchased from commercial suppliers and used without further purification. [Cu(MeCN)_4_]PF_6_, bis(diphenylphosphino)methane (dppm), 4-fluorophenylacetylene (HC≡CC_6_H_4_F), 4-chloroethynylbenzene (HC≡CC_6_H_4_Cl), 4-bromophenylacetylene (HC≡CC_6_H_4_Br) and 4-iodophenylacetylene (HC≡CC_6_H_4_I) were purchased from Titan, Shanghai, China.

### 3.1. Synthesis of Cu_3_-X

#### 3.1.1. Synthesis of the [Cu_3_(dppm)_3_(C≡CC_6_H_4_F)_2_]PF_6_ (**Cu_3_-F**)

To a solution of Cu(CH_3_CN)_4_PF_6_ (33.50 mg, 0.09 mmol), 4-fluorophenylacetylene (7.21 mg, 0.06 mmol), dppm (34.60 mg, 0.09 mmol), Et_3_N (30 μL), and DCM (4 mL) were charged. After stirring at room temperature overnight, the mixture was concentrated under reduced pressure. The residue was dissolved in DCM (2 mL) and filtered. White crystals suitable for single-crystals X-ray analysis can be obtained after one week by slow vapor diffusion of diethyl ether into the filtrate at ambient temperature. Yield: 36 mg, 69%, based on Cu(CH_3_CN)_4_PF_6_.^1^H NMR (400 MHz, CDCl_3_) δ 7.37–7.30 (m, 4H), 7.19 (t, *J* = 8.6 Hz, 4H), 7.10–7.01 (m, 36H), 6.82 (t, *J* = 7.7 Hz, 24H), 3.06 (s, 6H). ^31^P{^1^H} NMR (162 MHz, CDCl_3_) δ −5.37, −143.58. HR-ESI-MS calcd. for [Cu_3_(C_25_H_22_P_2_)_3_(C_8_H_4_F)_2_]^+^ 1582.2151, found 1582.6639; calcd. for [Cu_3_(C_25_H_22_P_2_)_2_(C_8_H_4_F)_2_]^+^ 1198.0953, found 1198.1968; calcd. for [Cu_2_(C_25_H_22_P_2_)_2_(C_8_H_4_F)]^+^ 1016.1360, found 1016.0740; calcd. for [Cu_3_(C_25_H_22_P_2_)(C_8_H_4_F)_2_]^+^ 813.9757, found 813.7292.

#### 3.1.2. Synthesis of the [Cu_3_(dppm)_3_(C≡CC_6_H_4_Cl)_2_]PF_6_ (**Cu_3_-Cl**)

The procedure was similar to that for **Cu_3_-F** except 4-chloroethynylbenzene (8.20 mg, 0.06 mmol) was used in place of 4-fluorophenylacetylene. White crystals of **Cu_3_-Cl** were obtained. Yield: 42 mg, 80%, based on Cu(CH_3_CN)_4_PF_6_. ^1^H NMR (400 MHz, CDCl_3_) δ 7.45 (d, *J* = 8.5 Hz, 4H), 7.27 (d, *J* = 8.4 Hz, 4H), 7.11–7.03 (m, 36H), 6.82 (t, *J* = 7.7 Hz, 24H), 3.07 (s, 6H). ^31^P{^1^H} NMR (162 MHz, CDCl_3_) δ −5.22, −143.60. HR-ESI-MS calcd. for [Cu_3_(C_25_H_22_P_2_)_3_(C_8_H_4_Cl)_2_]^+^ 1616.1559, found 1616.8998; calcd. for [Cu_3_(C_25_H_22_P_2_)_2_(C_8_H_4_Cl)_2_]^+^ 1230.0363, found 1230.3870; calcd. for [Cu_3_(C_25_H_22_P_2_)(C_8_H_4_Cl)_2_]^+^ 845.9166, found 845.8566.

#### 3.1.3. Synthesis of the [Cu_3_(dppm)_3_(C≡CC_6_H_4_Br)_2_]PF_6_ (**Cu_3_-Br**)

The procedure was similar to that for **Cu_3_-F** except 4-bromophenylacetylene (10.86 mg, 0.06 mmol) was used in place of 4-fluorophenylacetylene. Yellow block-shaped single crystals of **Cu_3_-Br** were obtained. Yield: 40 mg, 72%, based on Cu(CH_3_CN)_4_PF_6_. ^1^H NMR (400 MHz, CDCl_3_) δ 7.61 (d, *J* = 8.4 Hz, 4H), 7.20 (d, *J* = 8.4 Hz, 4H), 7.10–7.01 (m, 36H), 6.82 (t, *J* = 7.8 Hz, 24H), 3.06 (s, 6H). ^31^P{^1^H} NMR (162 MHz, CDCl_3_) δ −5.20, −143.62. HR-ESI-MS calcd. for [Cu_3_(C_25_H_22_P_2_)_3_(C_8_H_4_Br)_2_]^+^ 1704.0549, found 1704.6508; calcd. for [Cu_3_(C_25_H_22_P_2_)_2_(C_8_H_4_Br)_2_]^+^ 1319.9352, found 1320.1622; calcd. for [Cu_3_(C_25_H_22_P_2_)(C_8_H_4_Br)_2_]^+^ 935.8156, found 935.6915.

#### 3.1.4. Synthesis of the [Cu_3_(dppm)_3_(C≡CC_6_H_4_I)_2_]PF_6_ (**Cu_3_-I**)

The procedure was similar to that for **Cu_3_-F** except 4-iodophenylacetylene (13.68 mg, 0.06 mmol) was used in place of 4-fluorophenylacetylene. Yellow block-shaped single crystals of **Cu_3_-I** were obtained. Yield: 45 mg, 78%, based on Cu(CH_3_CN)_4_PF_6_. ^1^H NMR (400 MHz, CDCl_3_) δ 7.83 (d, *J* = 8.2 Hz, 4H), 7.12–7.02 (m, 40H), 6.84 (t, *J* = 7.6 Hz, 24H), 3.08 (s, 6H). ^31^P{^1^H} NMR (162 MHz, CDCl_3_) δ −5.78, −144.25. HR-ESI-MS calcd. for [Cu_3_(C_25_H_22_P_2_)_3_(C_8_H_4_I)_2_]^+^ 1798.0272, found 1798.6416; calcd. for [Cu_3_(C_25_H_22_P_2_)_2_(C_8_H_4_I)_2_]^+^ 1413.9075, found 1414.1814; calcd. for [Cu_3_(C_25_H_22_P_2_)(C_8_H_4_I)_2_]^+^ 1029.7878, found 1029.7231.

### 3.2. NMR and HR-ESI-MS Measurements

^1^H NMR spectra were recorded on a Bruker (Billerica, MA, USA) Biospin Avance III (400 MHz) spectrometer or JEOL (Akishima, Tokyo, Japan) ECZ400S/L1 spectrometer with chemical shifts (δ, ppm) relative to tetramethylsilane (Me_4_Si). COSY and DOSY NMR spectra were recorded on a Bruker Biospin Avance III (400 MHz) spectrometer. ^31^P{^1^H} NMR spectra were recorded on a Bruker Biospin Avance III 400 (162 MHz) Fourier transform NMR spectrometer with chemical shifts (δ, ppm) relative to 85% H_3_PO_4_.

HR-ESI-MS were recorded on a Waters (Milford, MA, USA) Synapt HDMS G2-Si mass spectrometer in positive reflection mode. The data analyses of mass spectra were performed based on the isotope distribution patterns using Waters MassLynx V4.1. The reported *m*/*z* values represent the monoisotopic mass of the most abundant peak within the isotope pattern.

### 3.3. Single-Crystal X-Ray Crystallography

X-Ray diffraction data of **Cu_3_-F**, **Cu_3_-Cl, Cu_3_-Br**, and **Cu_3_-I** were carried out on Synergy-R-Cu diffractometer equipped with a graphite-monochromated Cu-Kα radiation source (*λ* = 1.54184 Å) at 100 K. Using Olex2 (Version 1.5) [38], the crystal structures were determined by direct methods with ShelxT and refined by the full-matrix least-squares method based on F2 with the SHELXL 2016 [39,40,41]. Non-hydrogen atoms were refined anisotropically. All hydrogen atoms on C were bonded by theory. Some solvent molecules in the crystal structure were omitted using SQUEEZE program as they were highly disordered and could not be resolved unambiguously [42]. The X-ray crystallographic data for Cu_3_-X have been deposited at the Cambridge Crystallographic Data Centre (CCDC), number CCDC 2528138-2528141.

### 3.4. UV-Vis Absorption Spectra Measurements

The UV-vis absorption spectra of **Cu_3_-F**, **Cu_3_-Cl**, **Cu_3_-Br**, and **Cu_3_-I** was recorded using an Agilent (Santa Clara, CA, USA) Cary 60 UV-vis spectrophotometer using a quartz cuvette with a 1 cm optical path length. The data were collected in the wavelength range of 200–450 nm.

### 3.5. Optical Diffuse Reflectance Measurements

Optical diffuse reflectance spectra were measured by a Shimadzu (Tokyo, Japan) UV-2600i spectrophotometer with BaSO_4_ powder as the standard (100% reflectance) at room temperature. The data were collected in the wavelength range of 200–800 nm.

The relative absorbance (*A*) of the samples was converted from the diffuse reflectance data using the simplified formula:

A=−lg(R), 

where *R* is the relative reflectance of the sample calibrated against BaSO_4_.

In order to evaluate the band gap, the Kubelka–Munk function is used to collect and transform the data. Specifically, the Kubelka–Munk transformation converts the diffuse reflectance data (*R*_∞_) into the remission function *F*(*R*_∞_) using the equation:

$$F(R_{\infty })=\frac{{\left(1-R_{\infty }\right)}^{2}}{\left(2\times R_{\infty }\right)},\;$$

where *R*_∞_ represents the absolute reflectance of the sample relative to the BaSO_4_ reference.

The optical band gap (*E*_g_) was determined by plotting the Kubelka–Munk function *F*(*R*_∞_) against photon energy (*hν*, calculated as *hν* = 1240/*λ*, where *λ* is the wavelength in nm). A tangent line was fitted to the steepest linear portion of the absorption edge in the *F*(*R*_∞_) vs. *hν* plot, and the optical band gap was obtained by extrapolating this tangent to the *hν* axis at *F*(*R*_∞_) = 0.

### 3.6. Photoluminescence Spectra Measurements and Lifetime Measurements

Photoluminescence measurements of Cu_3_-X in the solid state were recorded using an Edinburgh Instrument (Livingston, UK) FLS1000 photoluminescence spectrometer at room temperature. The obtained sample powder was put into between two glass plates for measurements. The lifetime decay data is also recorded using an Edinburgh Instrument FLS1000 photoluminescence spectrometer at room temperature. The lifetimes of the sample were extracted by fitting the decay curves in Origin with first, second or third order exponential decay functions with coefficient of determination (*R*^2^) values larger than 0.99.

### 3.7. Quantum Yield Measurements

The room temperature absolute photoluminescence quantum yields of Cu_3_-X in powder were measured on an Edinburgh Instrument FLS1000 photoluminescence spectrometer with an integrating sphere accessory under ambient conditions. The sample to be measured is prepared by spreading the powder sample evenly on the bottom of the quartz sample holder.

### 3.8. Radiative and Non-Radiative Rate Constant

The radiative rate constant and non-radiative rate constant (*k*_r_ and *k*_nr_, respectively) were estimated by the following equations:

$$k_{r}=\frac{\Phi }{\tau },\;k_{nr}=\frac{1-\Phi }{\tau },$$

where *Φ* represents the absolute photoluminescence quantum yield of Cu_3_-X and the and *τ* represents its average photoluminescence lifetime.

### 3.9. Computational Details

All density functional theory (DFT) calculations were performed using the Vienna Ab initio Simulation Package (VASP, Version 6.0) [43,44,45], with the projector-augmented wave (PAW) method [46] to describe the electron-ion interactions. The Perdew-Burke-Ernzerhof (PBE) [47] functional within the generalized gradient approximation (GGA) was adopted for the exchange-correlation potential.

#### 3.9.1. Structural Optimization

The geometric optimization of the Cu cluster was carried out in a periodic cubic box with lattice parameters of 25.9675 × 25.9675 × 45.9675 Å^3^ (sufficient vacuum layer to eliminate periodic interactions between cluster images). The plane-wave cutoff energy was set to 500 eV to ensure the convergence of valence electron wavefunctions for both light (H/C/F) and heavy (Cu/I/Br/Cl/P) elements. The electronic self-consistency convergence criterion was set to 1 × 10^−6^ eV, and the ionic relaxation was terminated when the maximum Hellmann-Feynman force on each atom was less than 0.001 eV Å^−1^ (EDIFFG = −1 × 10^−3^). The conjugate gradient algorithm (IBRION = 2) was used for ionic relaxation with a maximum of 200 ionic steps (NSW = 200), and only atomic positions were optimized (ISIF = 2) while the lattice parameters were fixed. The real-space projection (LREAL = Auto) and augmented grid (ADDGRID = .TRUE.) were enabled to accelerate the calculation and improve the accuracy for heavy elements, respectively. Non-spherical pseudopotential correction (LASPH = .TRUE.) was adopted to enhance the precision of p-block elements (F/P).

#### 3.9.2. DOS and PDOS Calculations

After structural optimization, static self-consistent field (SCF) calculations were performed with a stricter electronic convergence criterion (1 × 10^−8^ eV) to generate accurate charge density (CHGCAR) and wavefunction (WAVECAR) files. Subsequently, non-self-consistent field (NSCF) calculations were carried out to compute the density of states (DOS) and projected density of states (PDOS), with the following key settings: the k-point sampling was restricted to the 1 × 1 × 1 Monkhorst-Pack grid due to the isolated cluster nature; the energy range for DOS sampling was set from −20 eV to 15 eV relative to the Fermi level, with 3000 discrete energy points (NEDOS = 3000) to ensure smooth DOS curves; the LORBIT = 11 tag was enabled to output full PDOS information (including s/p/d orbital projections for each atom).

The total DOS (TDOS) and PDOS data were extracted and visualized using VASPKIT [48], with the Fermi level set to zero for normalization. All calculations were performed on x-core CPU nodes with a parallelization scheme based on plane-wave and k-point decomposition.

#### 3.9.3. TD-DFT and SOC Calculations

All comprehensive excited-state TD-DFT calculations were performed using Gaussian 16 at the B3LYP-D3BJ level, with the LanL2DZ basis set for Cu, Br, and I atoms and 6-31G(d) for other atoms. SOC calculations were carried out using ORCA 6.0 at the B3LYP-D3BJ/cc-pvdz level.

## 4. Conclusions

In this study, a series of well-defined trinuclear copper(I) clusters protected by dppm and haloarylacetylene, [Cu_3_(dppm)_3_(C≡CC_6_H_4_X)_2_]PF_6_ (Cu_3_-X, X = F, Cl, Br, and I), were synthesized and systematically investigated. The luminescence mechanism of all Cu_3_-X clusters was confirmed to be triplet-state phosphorescence dominated by MLCT transitions with partial LLCT character, where the halogen p-orbitals (F 2p, Cl 3p, Br 4p, I 5p) participate in the construction of the conduction band, effectively regulating the excited-state energy levels and electronic transition properties of the clusters. The results demonstrate that the HAE introduced via halogen substitution on the ligands effectively modulates the luminescence properties of the clusters by enhancing the SOC strength and thus promoting the ISC process from S_1_ toT_1_. As the atomic number of the halogen increases, the emission peaks exhibit a pronounced red-shift from 491 nm for **Cu_3_-F** to 564 nm for **Cu_3_-I**, corresponding to a total shift of 73 nm. This redshift is attributed to the gradual decrease in the T_1_ energy induced by HAE, and this trend is consistent with the results of TD-DFT and SOC calculations. Meanwhile, both the phosphorescence quantum yield and lifetime are significantly enhanced for **Cu_3_-Cl** and **Cu_3_-Br**. **Cu_3_-Br** exhibited the optimal performance, with a 3.3-fold increase in quantum yield and a 3.7-fold extension in lifetime compared to **Cu_3_-F**. This superior luminescence performance is primarily due to the enhanced SOC that promotes ISC. It also suppresses non-radiative decay via a rigid crystal lattice and weak electron-phonon coupling. In contrast, **Cu_3_-I** shows a sharp drop in luminescence efficiency, arising from photoexcitation-induced structural instability as well as the synergistic effect of strong spin-vibration coupling and a soft lattice. These factors accelerate non-radiative recombination at room temperature and confirm the non-monotonic nature of the HAE. Excessive atomic size induces photodegradation and abnormal excited-state dynamics, which offset the benefits of enhanced ISC. This work clearly elucidates the role of ligand modification in regulating the photophysical properties of copper clusters via precise molecular design and structural characterization, and reveals the intrinsic links between halogen substitution, electronic structure, SOC strength and luminescence performance in Cu_3_ clusters, providing crucial guidance for the rational design of high-performance luminescent cluster-based materials and unraveling the structure-property relationship.

## Figures and Tables

**Table 1:** Emission peaks, estimated optical band gaps, luminescence lifetime, PLQY values, the radiative rate constant (*k*_r_) and non-radiative rate constant (*k*_nr_) of Cu_3_-X clusters.

Cluster Name	*λ*_em_ (nm)	CIE	Optical Band Gap (eV)	*τ* (μs, 298 K)	*τ* (μs, 77 K)	PLQY (%, 298 K)	*k*_r_ (×10^3^, s^−1^)	*k*_nr_ (×10^4^, s^−1^)
**Cu_3_-F**	491	(0.22, 0.43)	2.94	30.64	363.20	4.61	1.50	3.11
**Cu_3_-Cl**	502	(0.30, 0.56)	2.83	72.19	313.70	2.97	0.41	1.34
**Cu_3_-Br**	511	(0.35, 0.53)	2.79	114.56	313.70	15.24	1.33	0.74
**Cu_3_-I**	564	(0.44, 0.49)	2.84	18.08	264.84	0.19	0.11	5.52

## Data Availability

All data are contained within the article and its Supplementary Materials.

## Supplementary Materials

The following supporting information can be downloaded at: https://www.mdpi.com/article/10.3390/molecules31060987/s1, Figures S1–S16: ^1^H, ^31^P{^1^H},^1^H-^1^H COSY and DOSY NMR spectra of clusters; Figures S17–S20: HR-ESI-MS spectra of clusters; Figure S21: UV-Vis absorption spectra of ligands; Figures S22 and S23: UV-Vis diffuse reflectance spectra and Kubelka–Munk transformed spectra of clusters; Figure S24: Density of states plots; Figure S25: Plots of emission decay lifetime; Figure S26: Time-dependent evolution of ^1^H and ^31^P{1H} NMR spectra; Figures S27 and S28: Photoluminescence characteristics of Cu_3_-X clusters at 77 K; Tables S1–S12: Crystal data, structure refinement, selected bond lengths and angles for clusters; Table S13: Comparison of photophysical properties of Cu_3_-X clusters with literature reports; Table S14: Calculated triplet excitation energies, SOC constants, and emission wavelengths for Cu_3_-X clusters; Table S15: Molecular orbitals contributing to the T_1_ excited state of Cu_3_-X clusters. Refs. [20,33,34,36,37,49,50,51,52,53,54] are cited in the Supplementary Materials.

## Abbreviations

The following abbreviations are used in this manuscript:

HAE	Heavy-atom effect
ISC	Intersystem crossing
PLQY	Photoluminescence quantum yield
DOSY	Diffusion ordered spectroscopy
HR-ESI-MS	High-resolution electrospray-ionization mass spectrometry
dppm	bis(diphenylphosphino)methane
