# Supplementary material for: Heavy-Atom Effect Modulated Photoluminescence Properties of Trinuclear Copper(I) Clusters with Haloarylacetylene Ligands
Source: Molecules. 2026 Mar 15;31(6):987. doi: 10.3390/molecules31060987 (PMC13029245; doi:10.3390/molecules31060987)
Supplement: Supplementary file 1 [file molecules-31-00987-s001.zip › molecules-4182550-supplementary.pdf]

Supporting Information

Heavy-Atom Effect Modulated Photoluminescence Properties of Trinuclear Copper(I) Clusters with Haloarylacetylene Ligands

Qiao Yao [1,2], Ling-Ling Cai [2], Fang-Xue Xiao [1,2], Yu-Ting Ma [1,2], Shi-Yang Li [1,2], Jun Yi [2,*] and Yao Wang [2,*]

[1] College of Chemistry and Materials Science, Fujian Normal University, Fuzhou 350108, China; yaoqiao@fjirsm.ac.cn (Q.Y.); xiaofangxue@fjirsm.ac.cn (F.-X.X.); mayuting@fjirsm.ac.cn (Y.-T.M.); lisy@fjirsm.ac.cn (S.-Y.L.)

[2] State Key Laboratory of Structural Chemistry, Fujian Institute of Research on the Structure of Matter, Chinese Academy of Sciences, Fuzhou 350002, China; cailingling@fjirsm.ac.cn

* Correspondence: yijun@fjirsm.ac.cn (J.Y.); wangyao@fjirsm.ac.cn (Y.W.)

Contents

Figure S1. ^{1}H NMR (400 MHz, $CDCl_3$) spectrum of **Cu_3-F**. **S4**

Figure S2. $^{31}P\{^{1}H\}$ NMR (162 MHz, $CDCl_3$) spectrum of **Cu_3-F**. **S4**

Figure S3. ^{1}H NMR (400 MHz, $CDCl_3$) spectrum of **Cu_3-Cl**. **S5**

Figure S4. $^{31}P\{^{1}H\}$ NMR (162 MHz, $CDCl_3$) spectrum of **Cu_3-Cl**. **S5**

Figure S5. ^{1}H NMR (400 MHz, $CDCl_3$) spectrum of **Cu_3-Br**. **S6**

Figure S6. $^{31}P\{^{1}H\}$ NMR (162 MHz, $CDCl_3$) spectrum of **Cu_3-Br**. **S6**

Figure S7. ^{1}H NMR (400 MHz, $CDCl_3$) spectrum of **Cu_3-I**. **S7**

Figure S8. $^{31}P\{^{1}H\}$ NMR (162 MHz, $CDCl_3$) spectrum of **Cu_3-I**. **S7**

Figure S9. ^{1}H-^{1}H COSY NMR (400 MHz, $CDCl_3$) spectrum of **Cu_3-F**. **S8**

Figure S10. ^{1}H-^{1}H COSY NMR (400 MHz, $CDCl_3$) spectrum of **Cu_3-Cl**. **S8**

Figure S11. ^{1}H-^{1}H COSY NMR (400 MHz, $CDCl_3$) spectrum of **Cu_3-Br**. **S9**

Figure S12. ^{1}H-^{1}H COSY NMR (400 MHz, $CDCl_3$) spectrum of **Cu_3-I**. **S9**

Figure S13. ^{1}H DOSY NMR (400 MHz, $CDCl_3$) spectrum of **Cu_3-F**. **S10**

Figure S14. ^{1}H DOSY NMR (400 MHz, $CDCl_3$) spectrum of **Cu_3-Cl**. **S10**

Figure S15. ^{1}H DOSY NMR (400 MHz, $CDCl_3$) spectrum of **Cu_3-Br**. **S11**

Figure S16. ^{1}H DOSY NMR (400 MHz, $CDCl_3$) spectrum of **Cu_3-I**. **S11**

Figure S17. HR-ESI-MS spectra of **Cu_3-F**. **S12**

Figure S18. HR-ESI-MS spectra of **Cu_3-Cl**. **S12**

Figure S19. HR-ESI-MS spectra of **Cu_3-Br**. **S13**

Figure S20. HR-ESI-MS spectra of **Cu_3-I**. **S13**

Figure S21. UV-Vis absorption spectra of ligands **S13**

Figure S22. UV-Vis diffuse reflectance spectra of Cu_3-X. **S14**

Figure S23. Kubelka–Munk transformed spectra. **S14**

Figure S24. The density of states plots for Cu_3-X. **S15**

Figure S25. Plots of emission decay lifetime of Cu_3-X at room temperature. **S15**

Figure S26. Time-dependent evolution of (a) ^{1}H and (b) $^{31}P\{^{1}H\}$ NMR spectra of **Cu_3-I** upon irradiation with 365 nm UV light. **S16**

Figure S27. Excitation and emission spectra of Cu_3-X at 77 K. **S16**

Figure S28. Plots of emission decay lifetime of Cu_3-X at 77 K. **S17**

Table S1. Crystal data and structure refinement of **Cu_3-F**. **S18**

Table S2. Selected bond lengths (Å) for **Cu_3-F**. **S19**

Table S3. Selected bond angles (°) for **Cu_3-F**. **S20**

Table S4. Crystal data and structure refinement of **Cu_3-Cl**. **S21**

Table S5. Selected bond lengths (Å) for **Cu_3-Cl**. **S22**

Table S6. Selected bond angles (°) for **Cu_3-Cl**. **S23**

Table S7. Crystal data and structure refinement of **Cu_3-Br**. **S24**

Table S8. Selected bond lengths (Å) for **Cu_3-Br**. **S25**

Table S9. Selected bond angles (°) for **Cu_3-Br**. **S26**

Table S10. Crystal data and structure refinement of **Cu_3-I**. **S27**

Table S11. Selected bond lengths (Å) for **Cu_3-I**. **S28**

Table S12. Selected bond angles (°) for **Cu_3-I**. **S29**

Table S13. Photophysical properties of copper(I) cluster complexes reported in the literature and this work. **S30**

Table S14. Calculated Triplet Excitation Energies ($\Delta E(S_0 \rightarrow T_1)$), Spin-Orbit Coupling (SOC) Constants between S_1 and T_1, Calculated Emission Wavelengths (Cal. λ_{em}), and Experimental Emission Wavelengths (Expt. λ_{em}) for Cu_3-X Clusters. **S31**

Table S15. Molecular orbitals contributing to the T_1 excited state (responsible for $T_1 \rightarrow S_0$ phosphorescence) of Cu_3-X Clusters. **S31**

Reference **S32**

Supplementary Figures and Tables

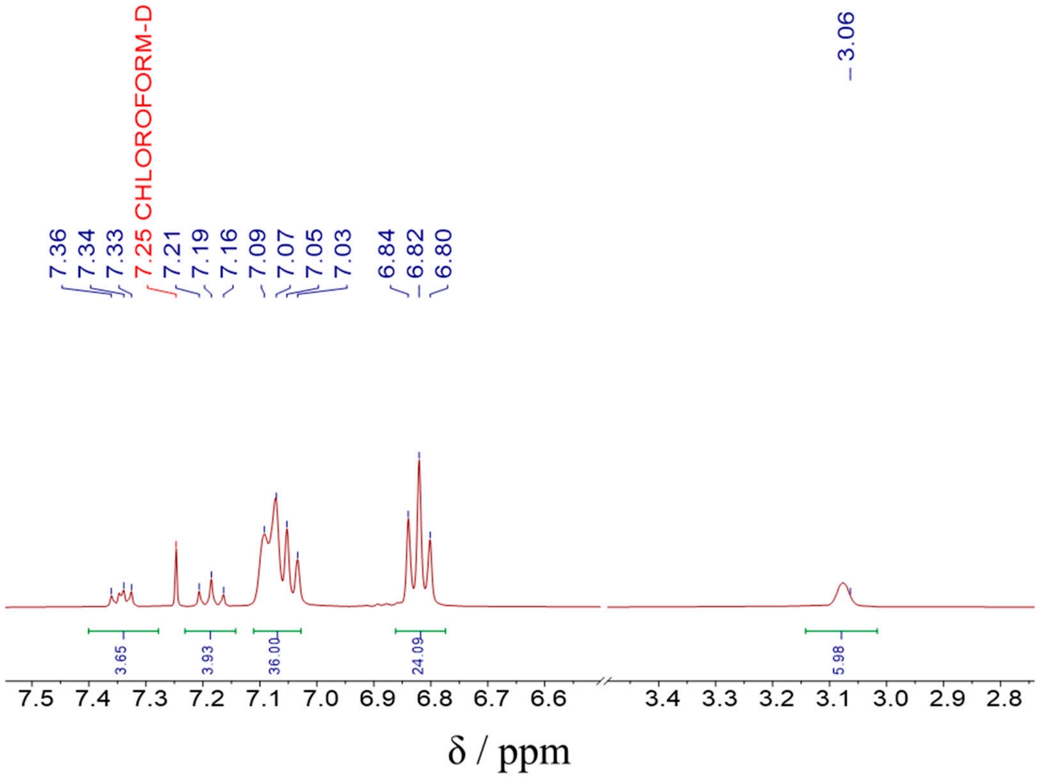

Figure S1. ^{1}H NMR (400 MHz, $CDCl_3$) spectrum of **Cu_3-F**.

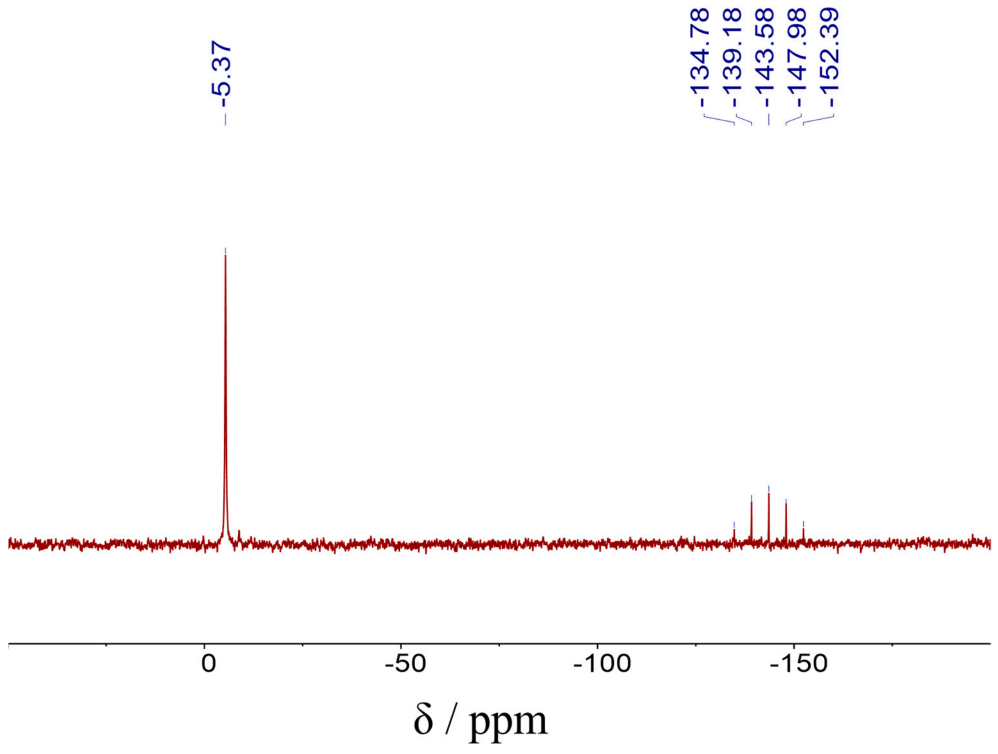

Figure S2. $^{31}P\{^{1}H\}$ NMR (162 MHz, $CDCl_3$) spectrum of **Cu_3-F**.

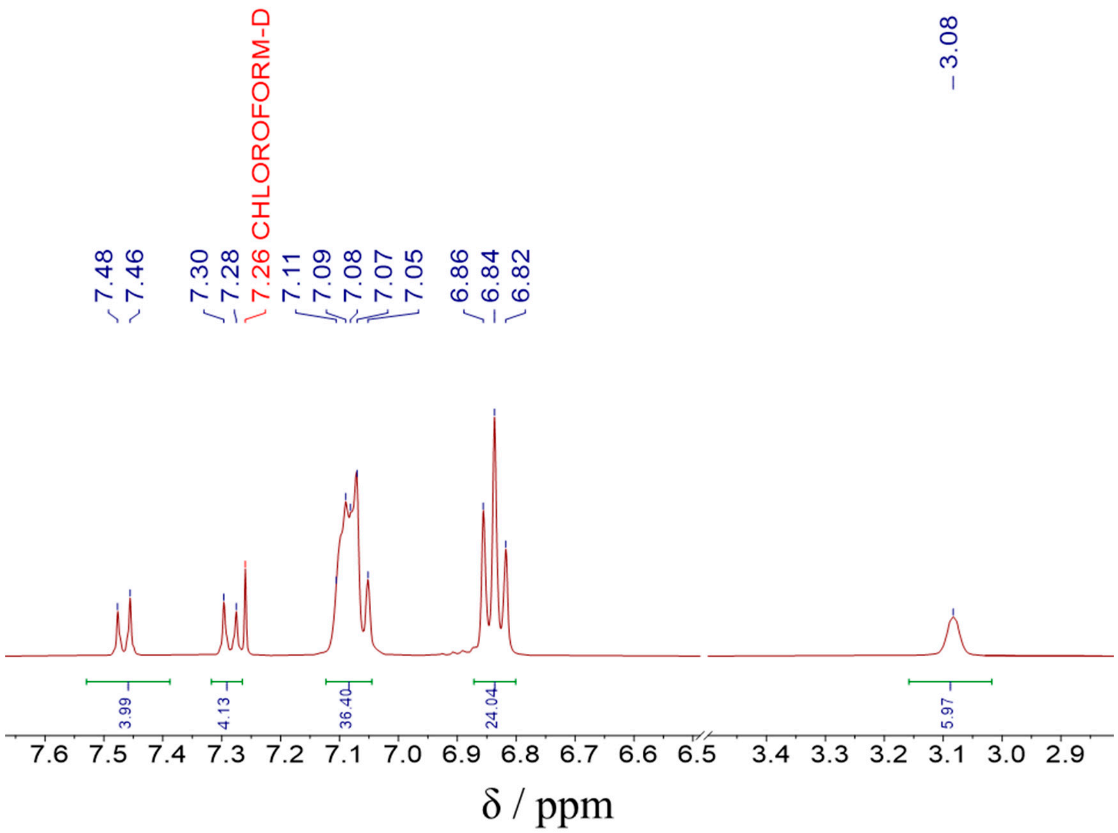

Figure S3. ^{1}H NMR (400 MHz, $CDCl_3$) spectrum of **Cu_3-Cl**.

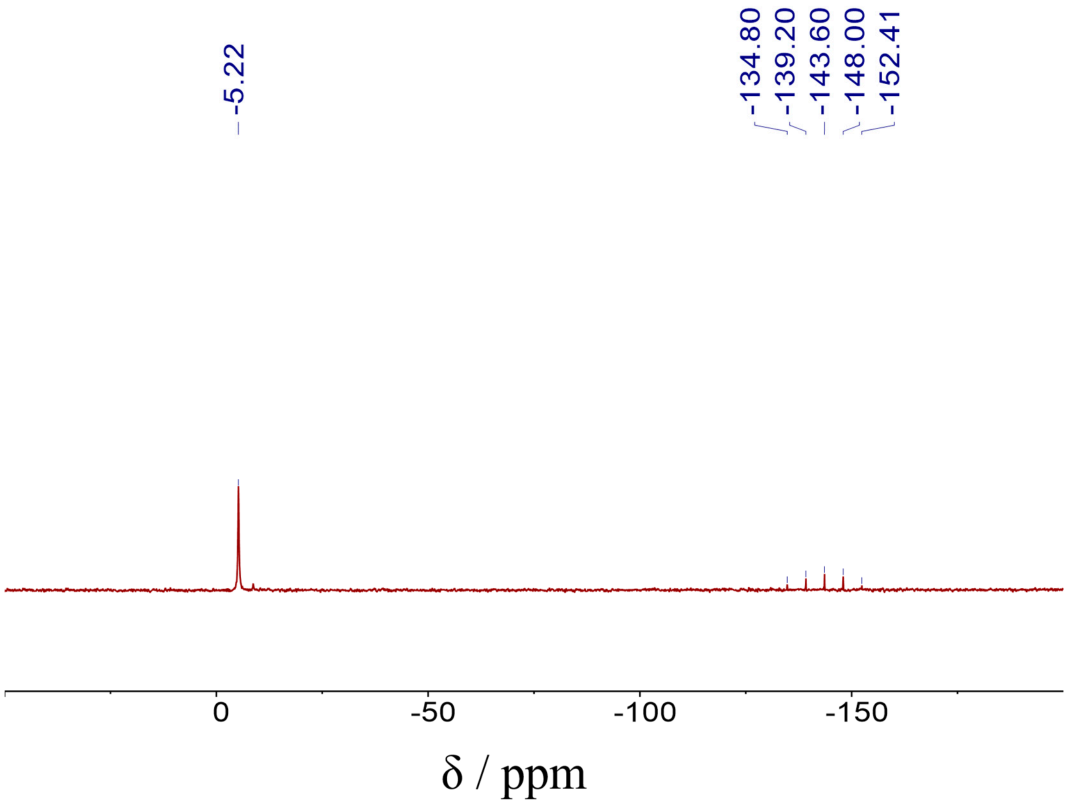

Figure S4. $^{31}P\{^{1}H\}$ NMR (162 MHz, $CDCl_3$) spectrum of **Cu_3-Cl**.

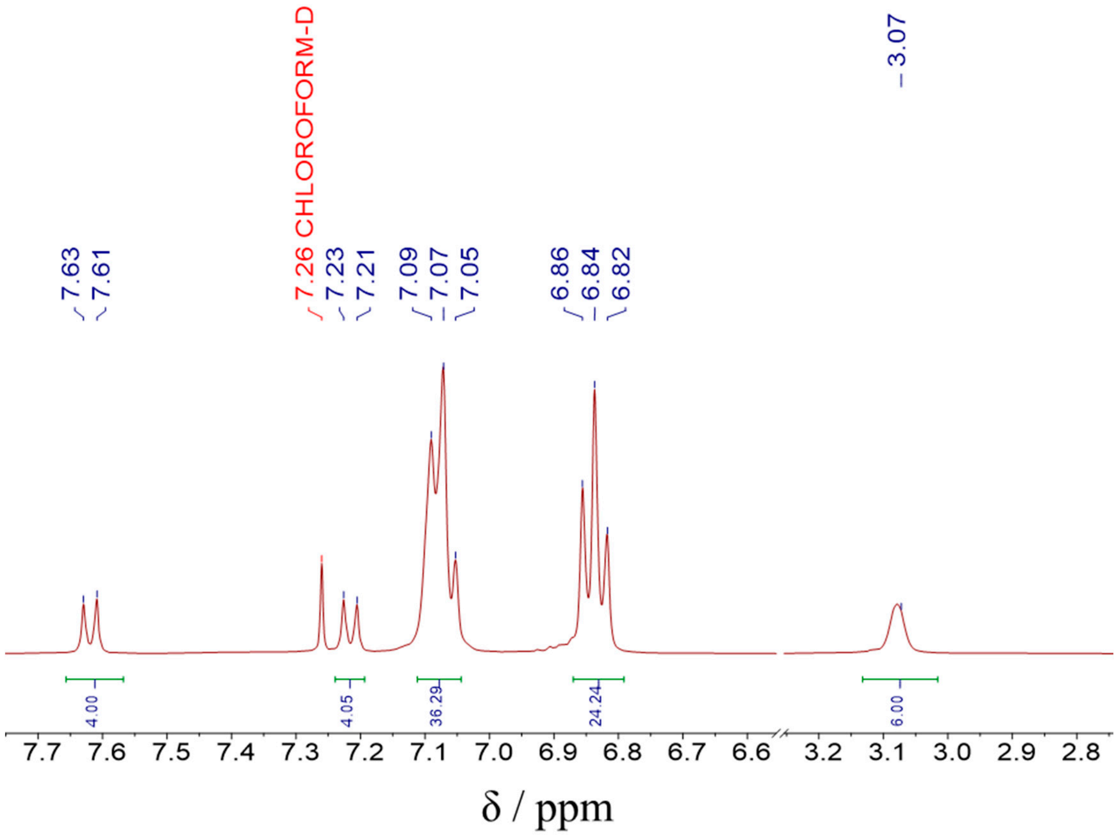

Figure S5. ^{1}H NMR (400 MHz, $CDCl_3$) spectrum of **Cu_3-Br**.

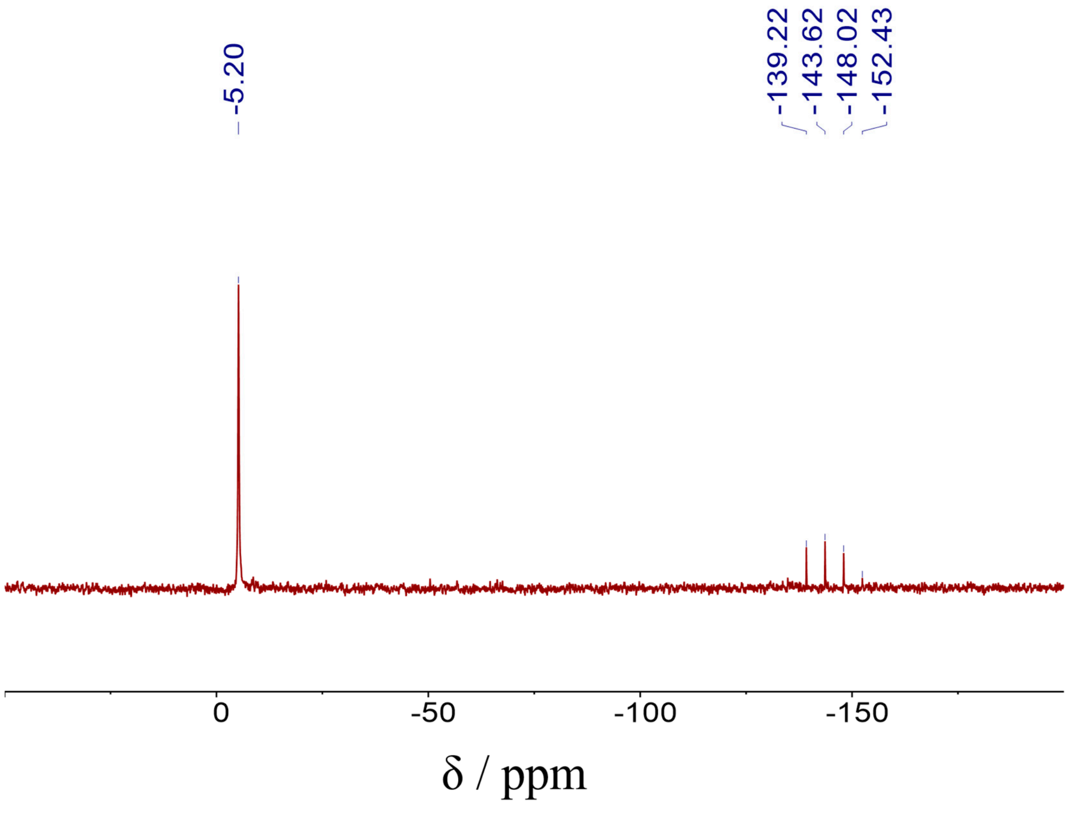

Figure S6. $^{31}P\{^1H\}$ NMR (162 MHz, $CDCl_3$) spectrum of **Cu_3-Br**.

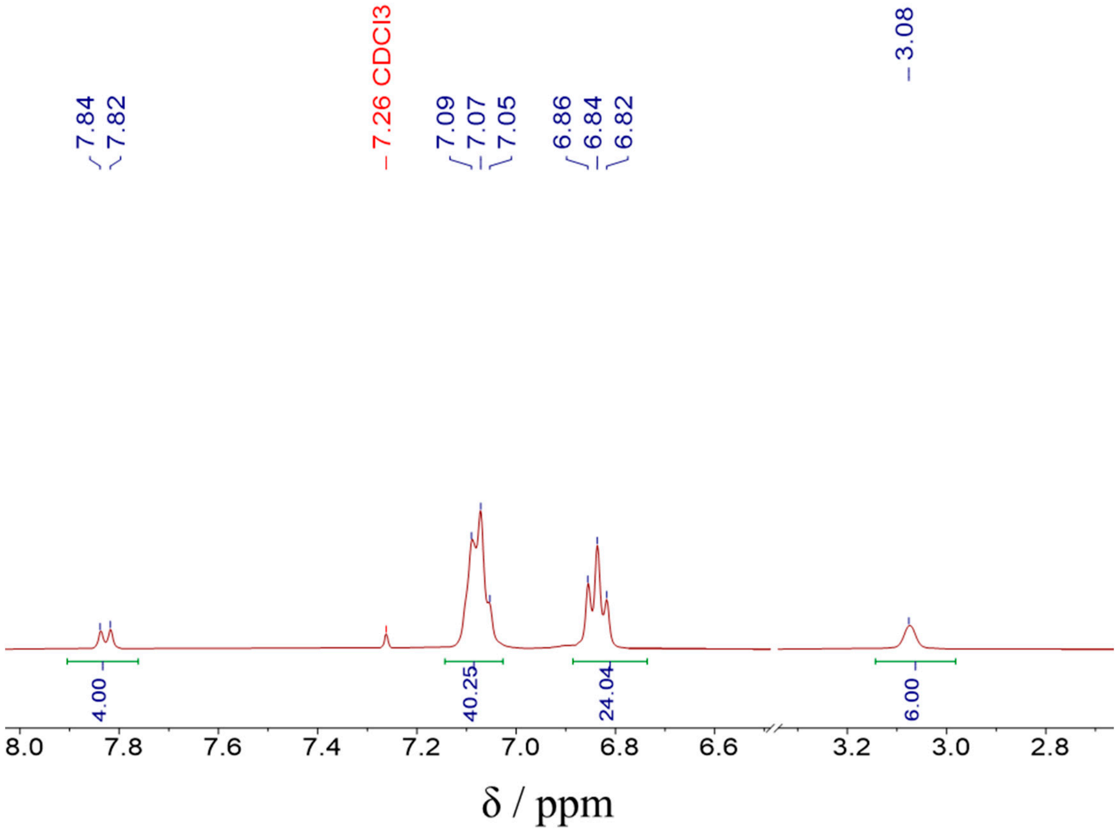

Figure S7. ^{1}H NMR (400 MHz, $CDCl_3$) spectrum of **Cu_3-I**.

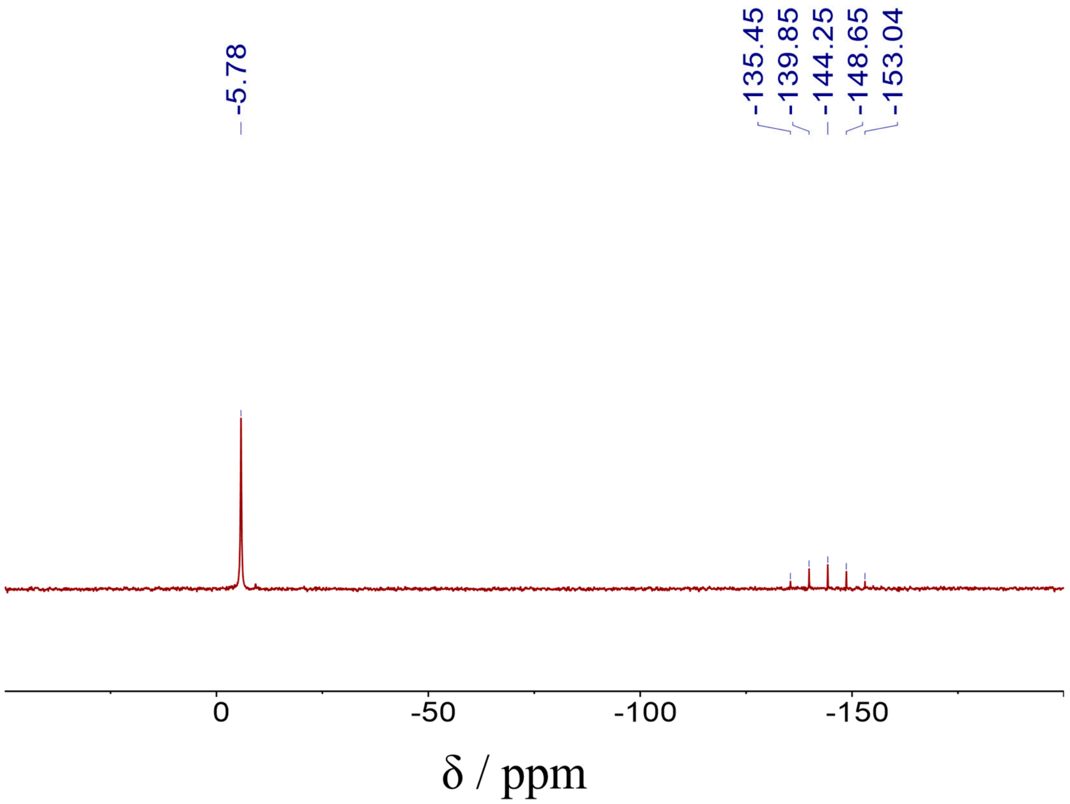

Figure S8. $^{31}P\{^{1}H\}$ NMR (162 MHz, $CDCl_3$) spectrum of **Cu_3-I**.

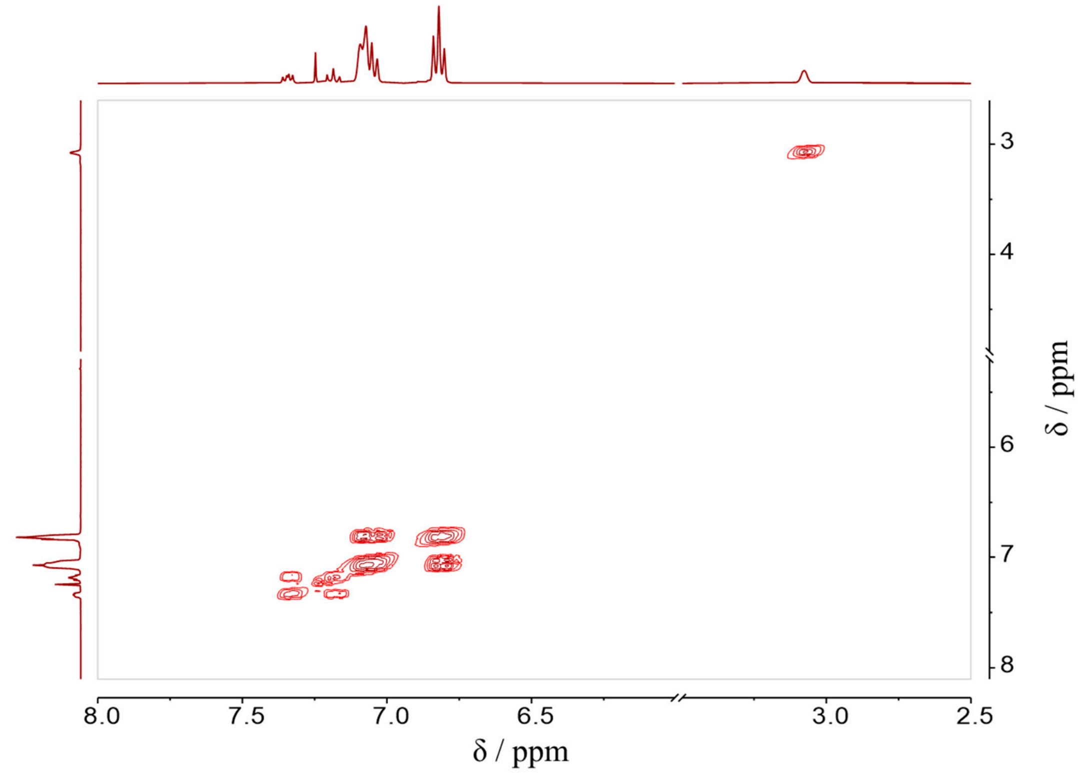

Figure S9. ^{1}H-^{1}H COSY NMR (400 MHz, $CDCl_3$) spectrum of **Cu_3-F**.

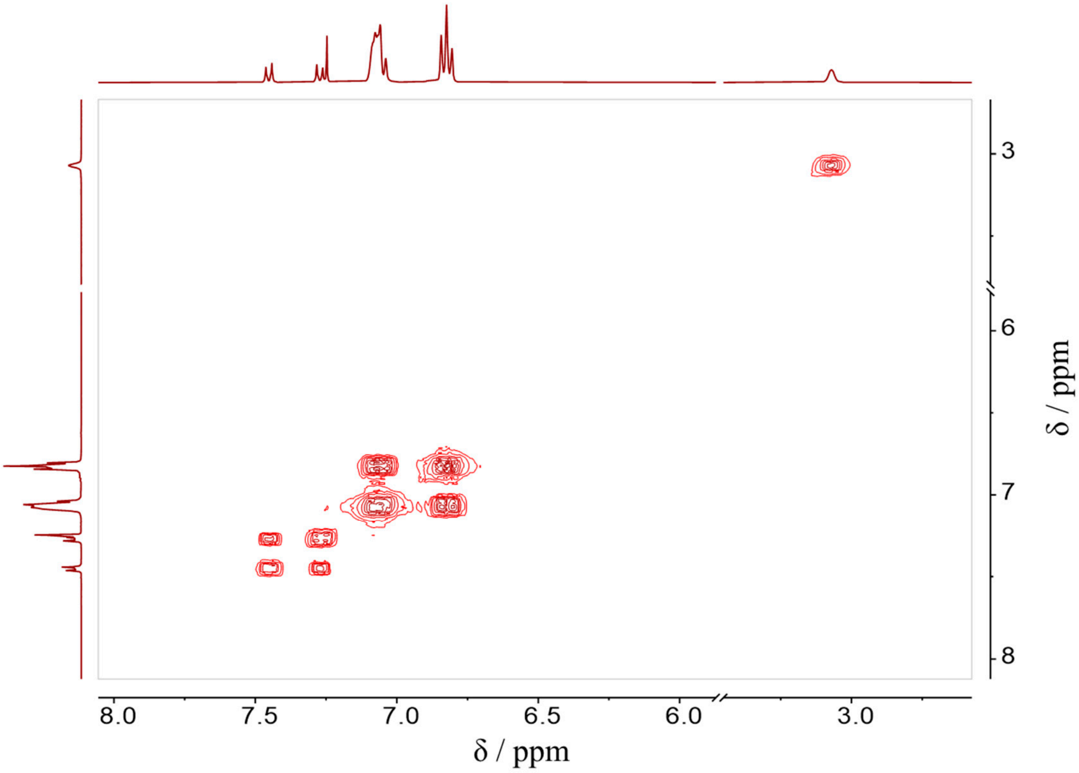

Figure S10. ^{1}H-^{1}H COSY NMR (400 MHz, $CDCl_3$) spectrum of **Cu_3-Cl**.

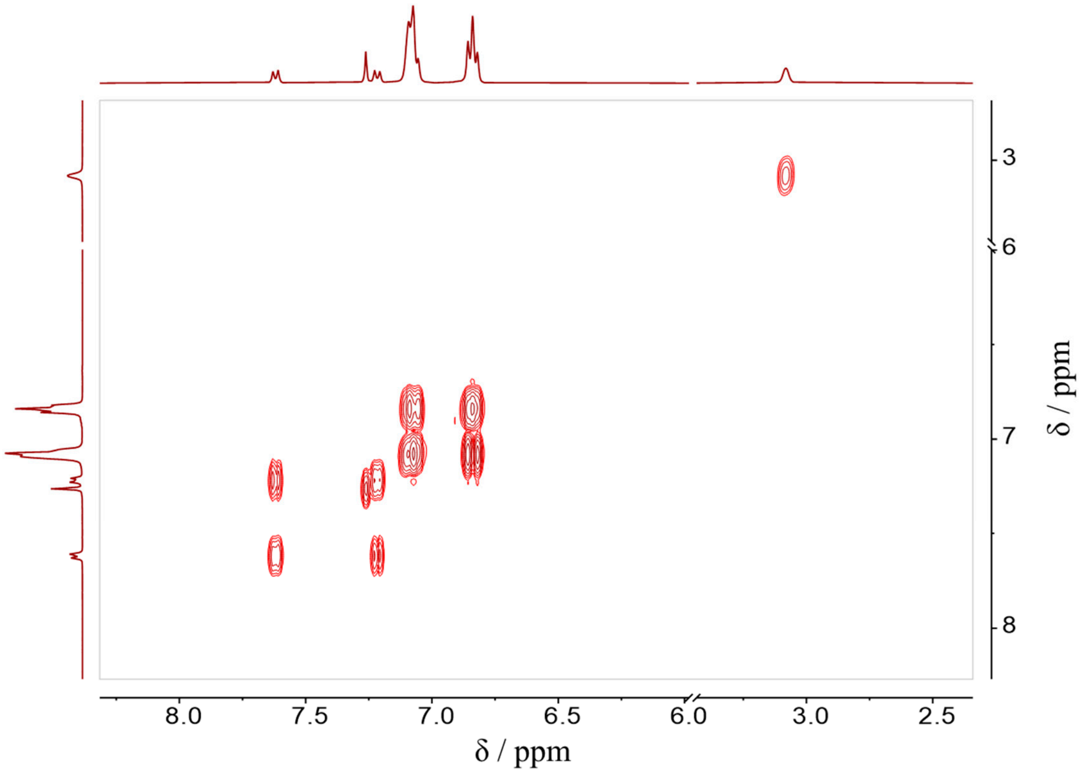

Figure S11. ^{1}H-^{1}H COSY NMR (400 MHz, $CDCl_3$) spectrum of **Cu_3-Br**.

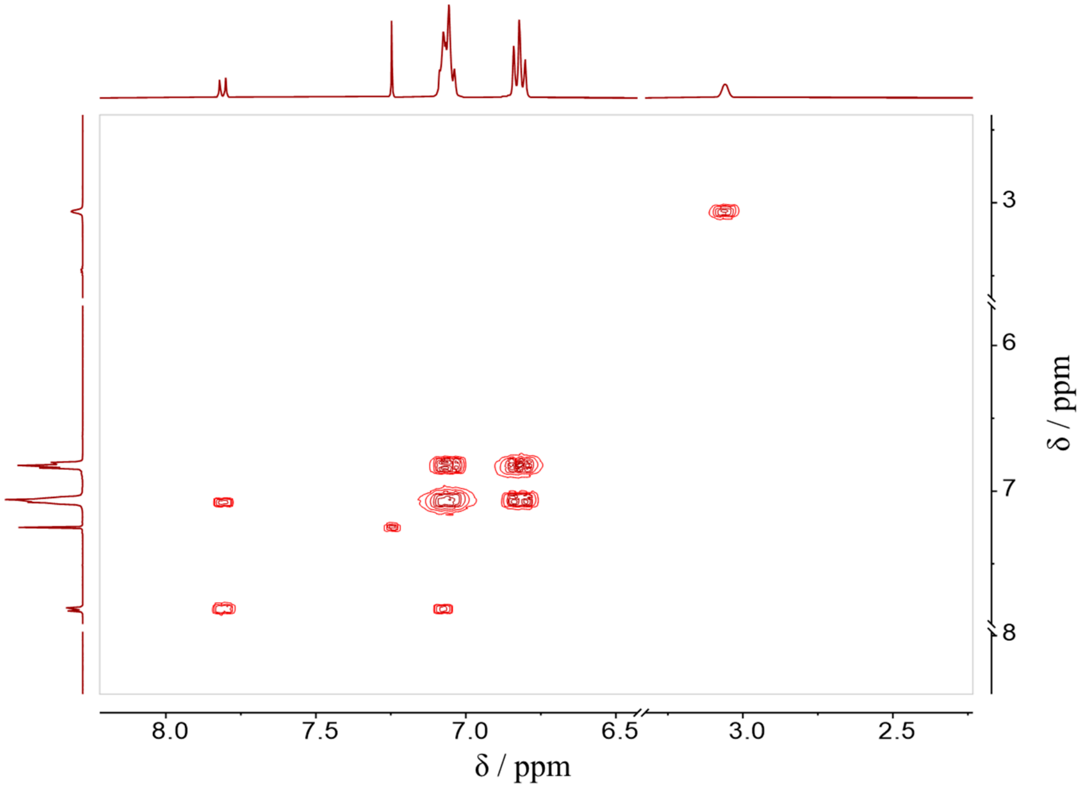

Figure S12. ^{1}H-^{1}H COSY NMR (400 MHz, $CDCl_3$) spectrum of **Cu_3-I**.

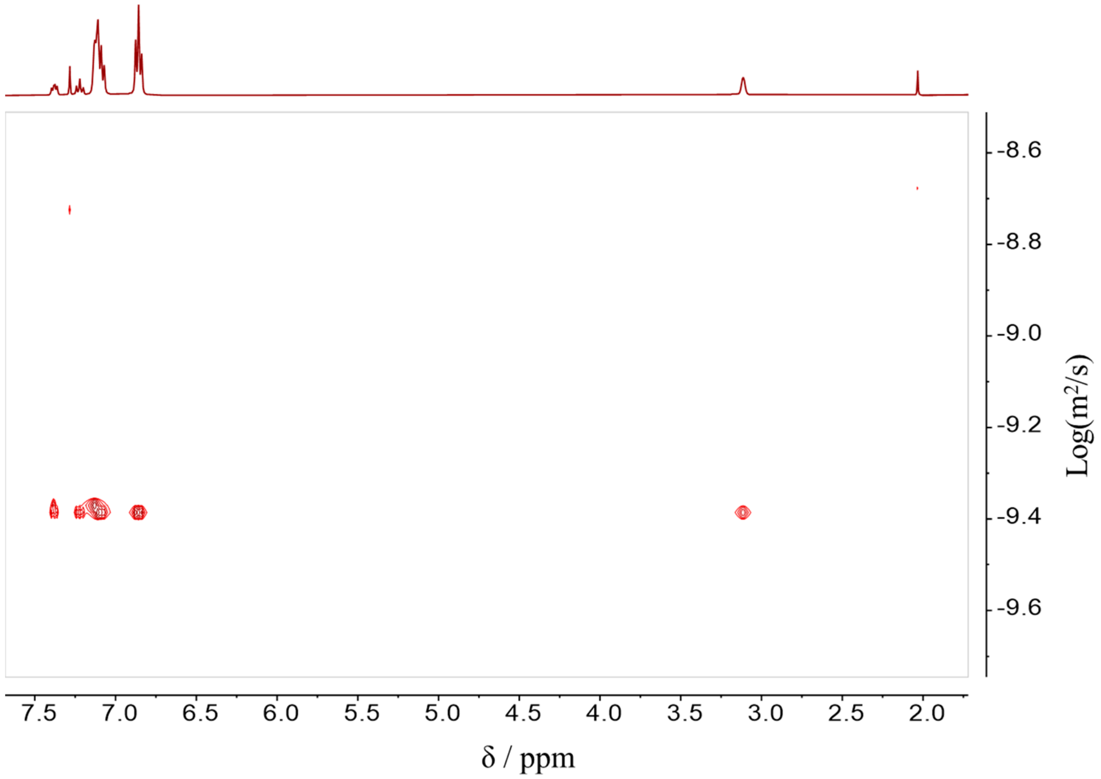

Figure S13. ^{1}H DOSY NMR (400 MHz, $CDCl_3$) spectrum of **Cu_3-F**.

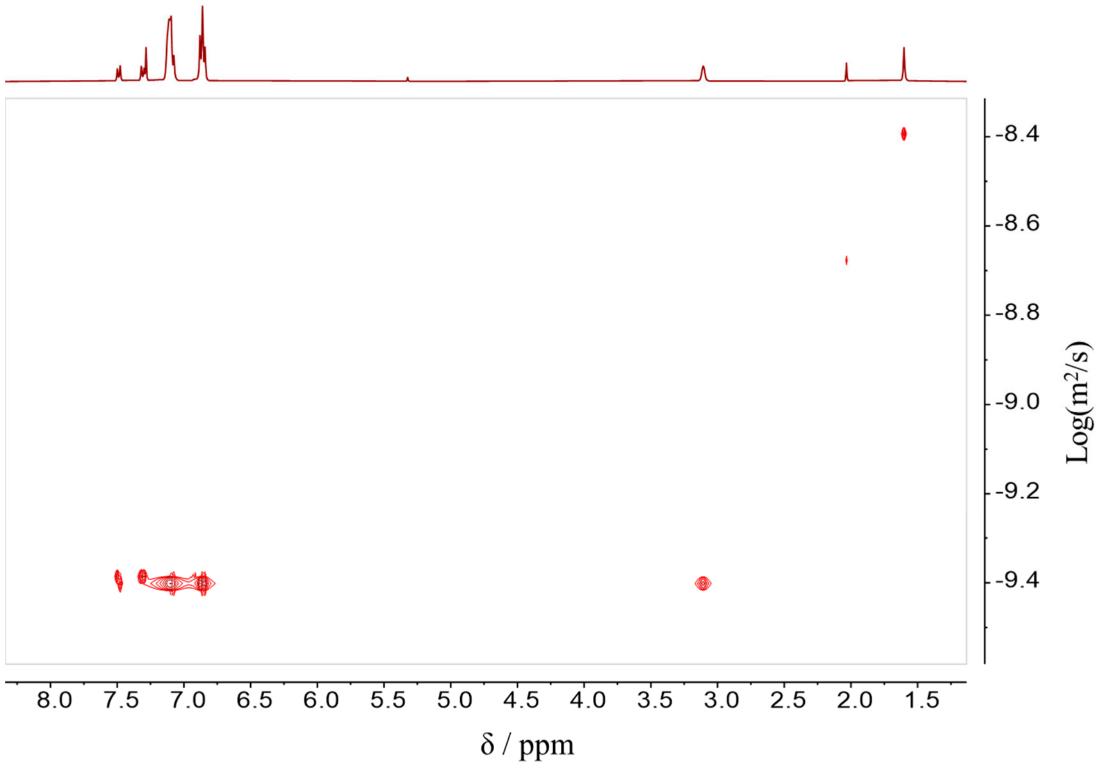

Figure S14. ^{1}H DOSY NMR (400 MHz, $CDCl_3$) spectrum of **Cu_3-Cl**.

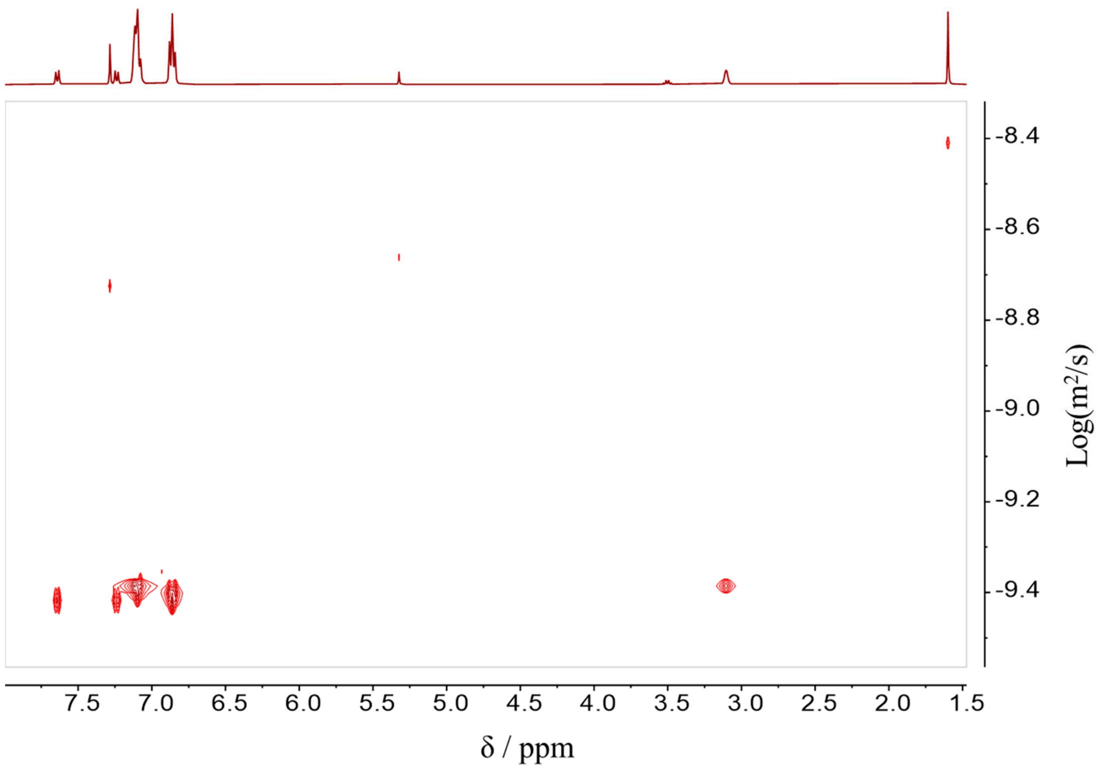

Figure S15. ^{1}H DOSY NMR (400 MHz, $CDCl_3$) spectrum of **Cu_3-Br**.

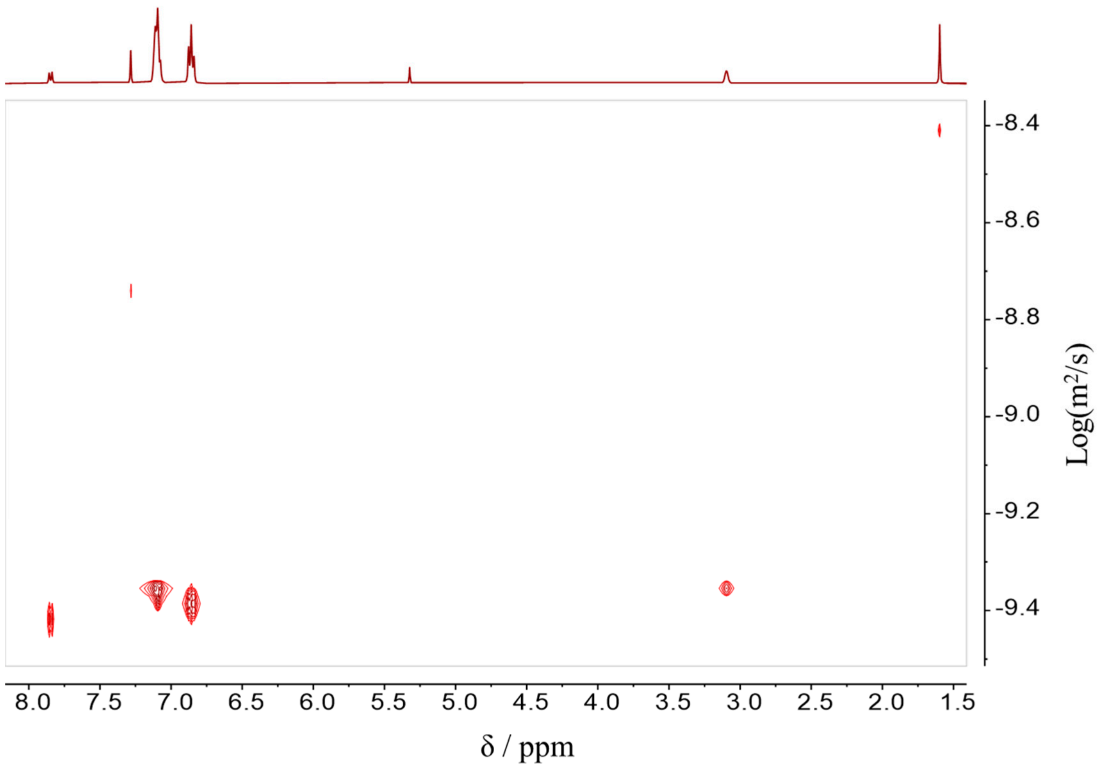

Figure S16. ^{1}H DOSY NMR (400 MHz, $CDCl_3$) spectrum of **Cu_3-I**.

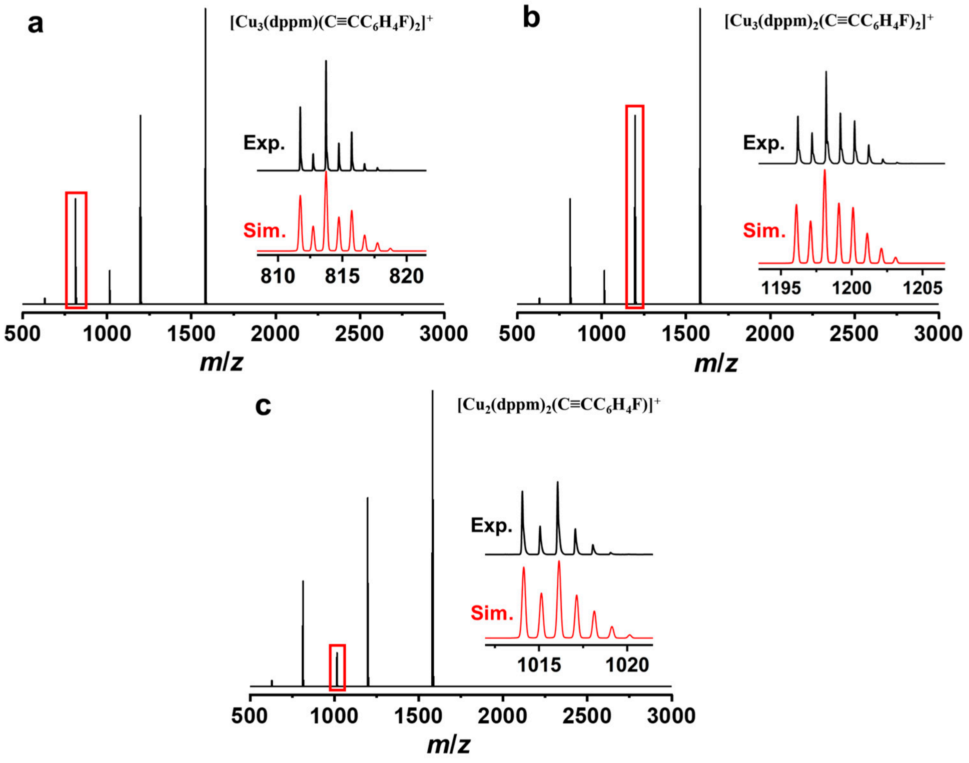

Figure S17. HR-ESI-MS spectra of **Cu3-F** with insets showing a comparison between the experimental (Exp.) and simulated (Sim.) isotope patterns for the component signals.

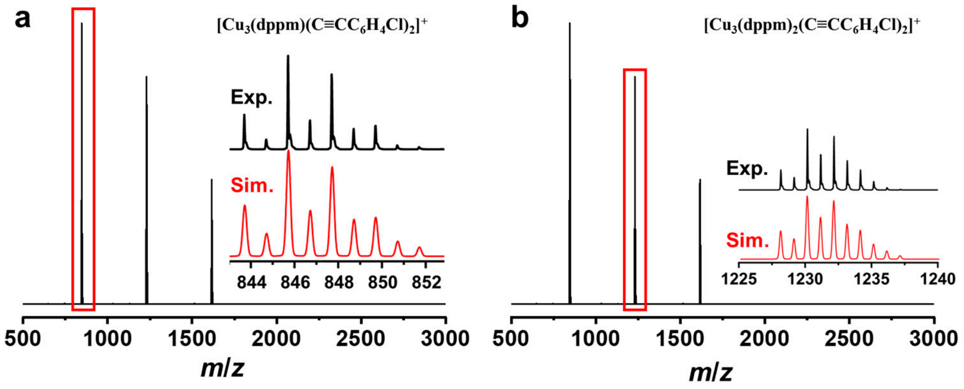

Figure S18. HR-ESI-MS spectra of **Cu3-Cl** with insets showing a comparison between the experimental (Exp.) and simulated (Sim.) isotope patterns for the component signals.

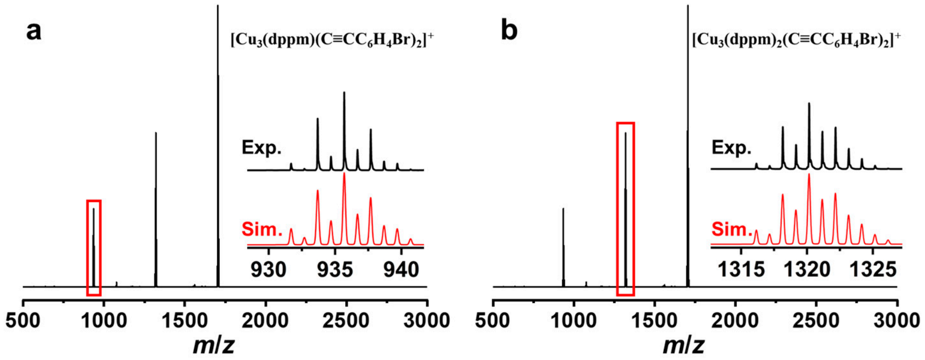

Figure S19. HR-ESI-MS spectra of **Cu_3-Br** with insets showing a comparison between the experimental (Exp.) and simulated (Sim.) isotope patterns for the component signals.

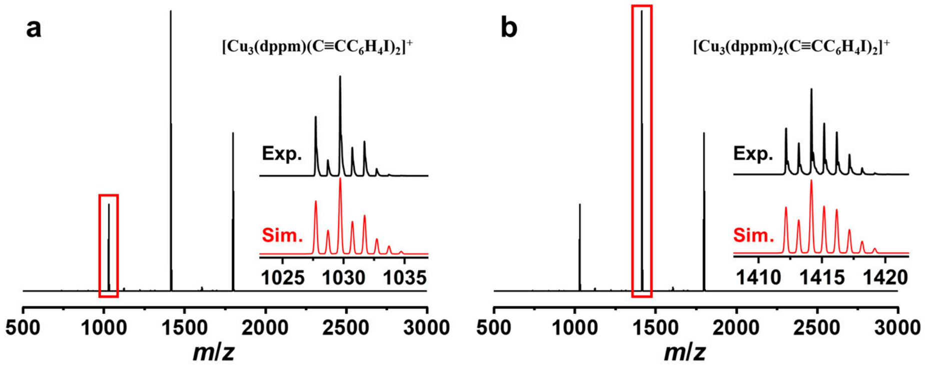

Figure S20. HR-ESI-MS spectra of **Cu_3-I** with insets showing a comparison between the experimental (Exp.) and simulated (Sim.) isotope patterns for the component signals.

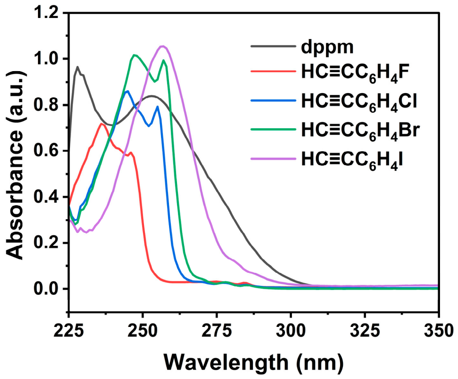

Figure S21. UV-Vis absorption spectra of dppm (conc. 1×10^{-5} mol L^{-1}) and $HC \equiv CC_6H_4X$ (X = F, Cl, Br, and I) (conc. 4×10^{-5} mol L^{-1}) in CH_2Cl_2.

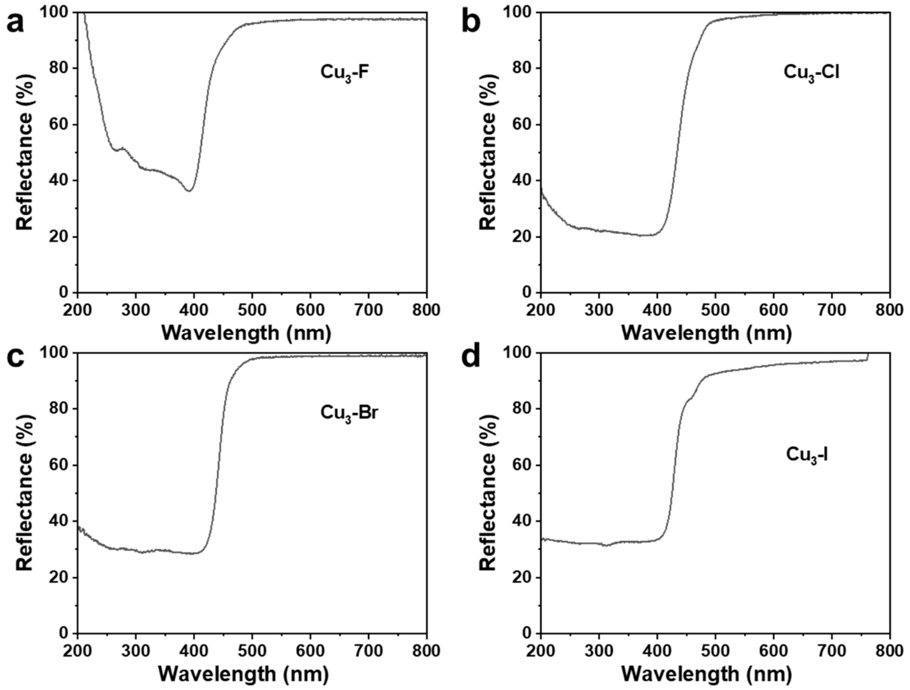

Figure S22. UV-Vis diffuse reflectance spectra of Cu_3-X (X = F, Cl, Br, and I).

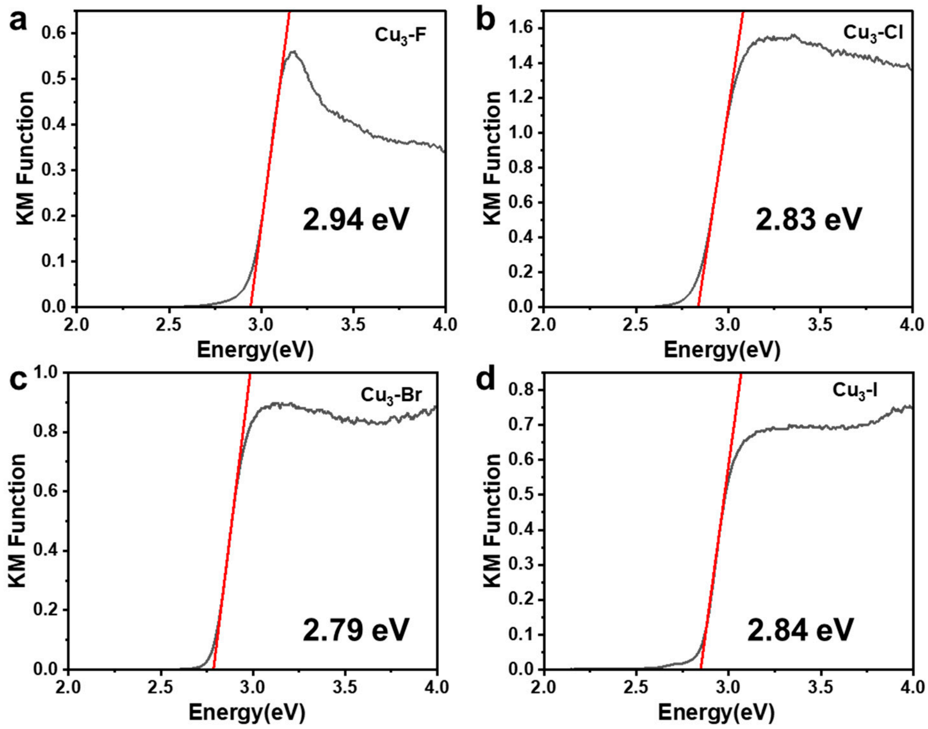

Figure S23. Kubelka–Munk transformed spectra. The optical band gap E_g was obtained by linearly fitting the steepest rising segment of the absorption edge and extrapolating the tangent line to intersect the photon energy axis at $F(R_\infty) = 0$.

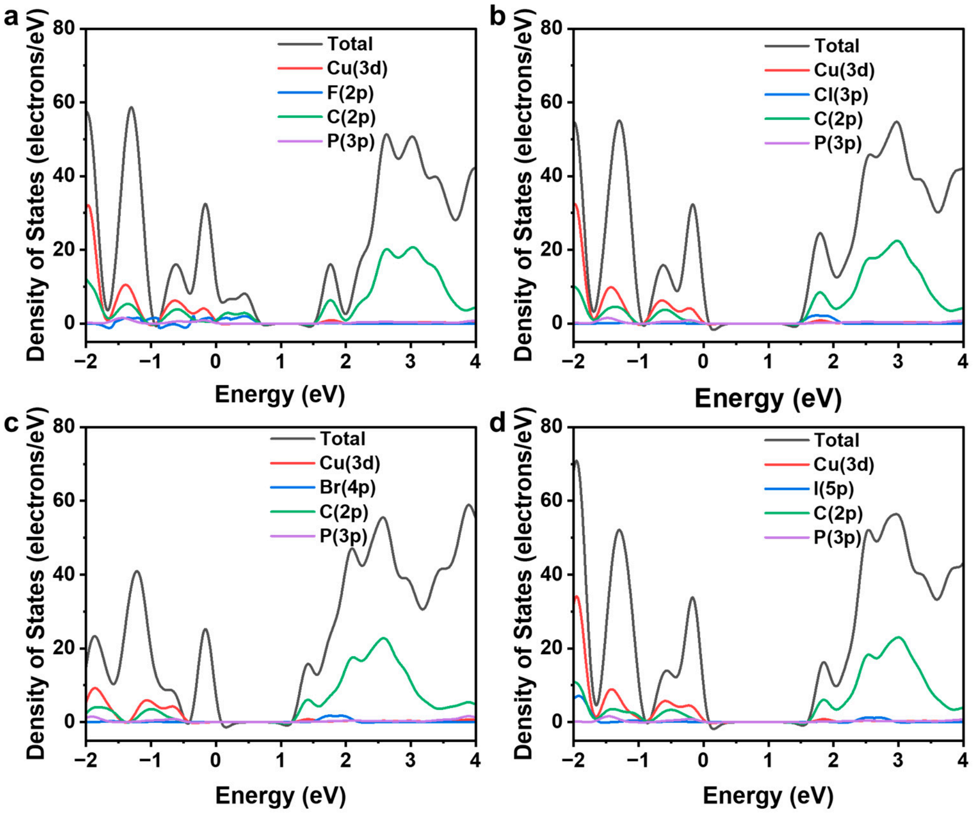

Figure S24. The total density of states and projected density of states plots for (a) **Cu_3-F**, (b) **Cu_3-Cl**, (c) **Cu_3-Br**, and (d) **Cu_3-I**.

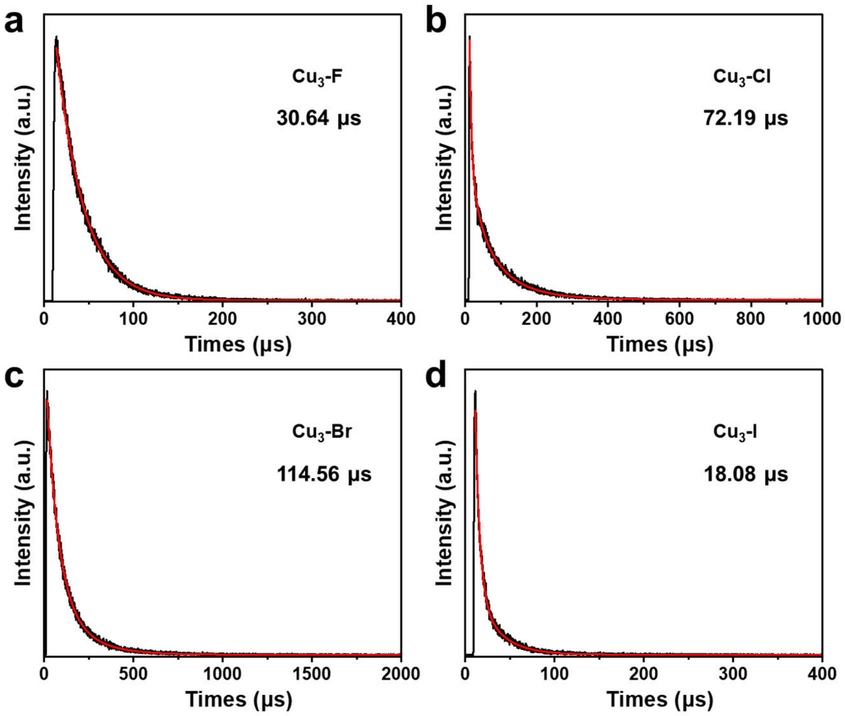

Figure S25. Plots of emission decay lifetime of Cu_3-X at room temperature.

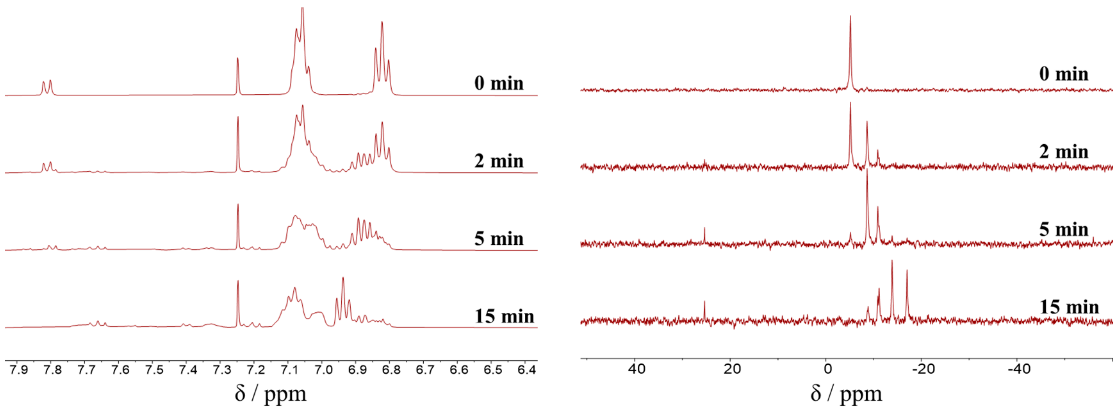

Figure S26. Time-dependent evolution of (a) ^{1}H and (b) $^{31}P\{^{1}H\}$ NMR spectra of **Cu_3-I** upon irradiation with 365 nm UV light.

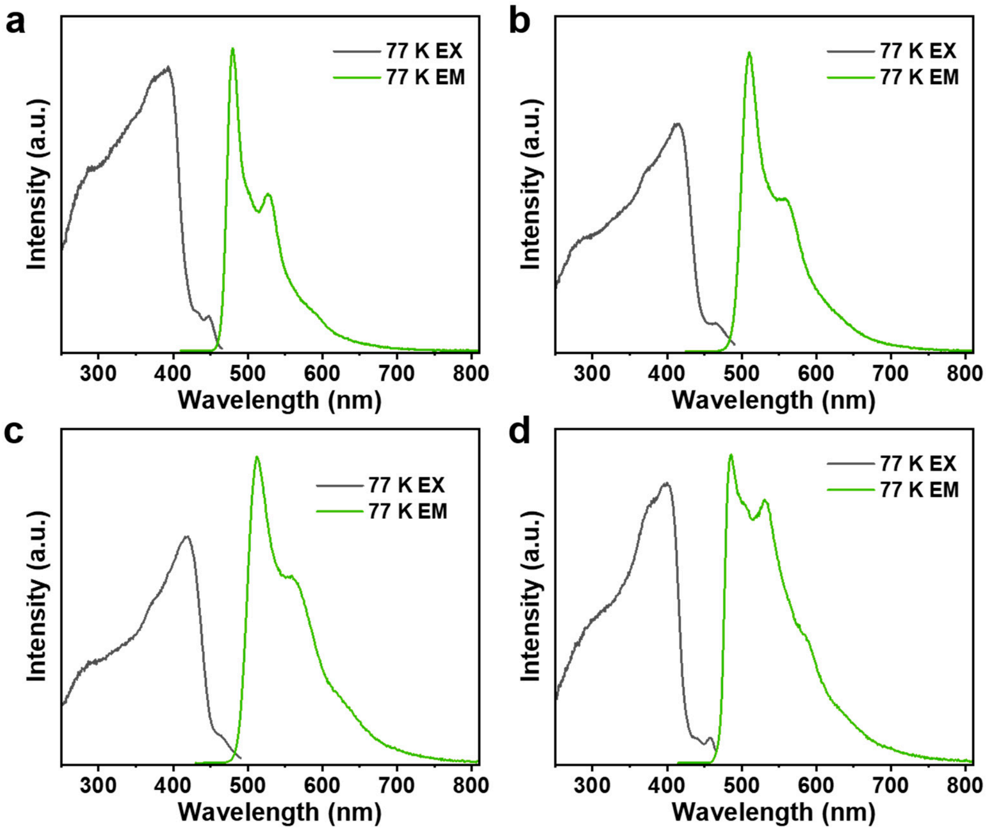

Figure S27. Excitation (black) and emission (green) spectra of (a) **Cu_3-F**, (b) **Cu_3-Cl**, (c) **Cu_3-Br**, and (d) **Cu_3-I** at 77 K.

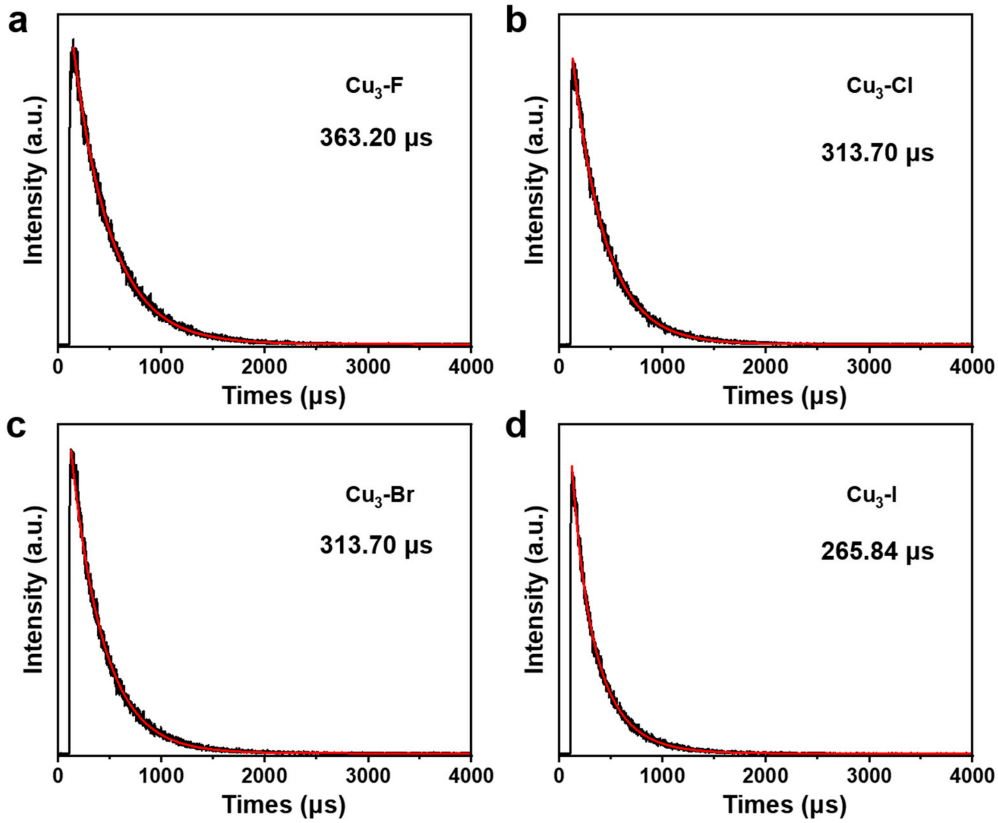

Figure S28. Plots of emission decay lifetime of Cu_3-X at 77 K

Table S1. Crystal data and structure refinement of **Cu_3-F**.

Identification code	Cu_3-F
Empirical formula	$C_{91}H_{74}Cu_3F_2P_6$
Formula weight	1581.94
Temperature/K	100.00(10)
Crystal system	monoclinic
Space group	Cc
a/Å	14.16480(10)
b/Å	25.5387(2)
c/Å	25.1371(3)
α/°	90
β/°	95.7590(10)
γ/°	90
Volume/Å^3	9047.46(14)
Z	4
ρ_{calc}g/cm^3	1.161
μ/mm^{-1}	2.163
F(000)	3260
Crystal size/mm^3	0.8 × 0.3 × 0.1
Radiation	Cu Kα (λ = 1.54184)
2θ range for data collection/°	6.922 to 152.358
Index ranges	$-17 \leq h \leq 17$, $-32 \leq k \leq 29$, $-29 \leq l \leq 31$
Reflections collected	30581
Independent reflections	14195 [R_{int} = 0.0198, R_{sigma} = 0.0221]
Data/restraints/parameters	14195/2/920
Goodness-of-fit on F^2	1.027
Final R indexes [I>=2σ (I)]	R_1 = 0.0260, wR_2 = 0.0678
Final R indexes [all data]	R_1 = 0.0262, wR_2 = 0.0680
Largest diff. peak/hole / e Å^{-3}	0.31/−0.37
Flack parameter	0.171(11)

Table S2. Selected bond lengths (Å) for **Cu$_3$-F**.

P2−C13	1.827(3)	C61−C62	1.399(5)
P2−C25	1.823(3)	C97−C98	1.394(4)
P2−C11	1.837(3)	C97−C96	1.402(4)
P2−Cu3	2.3047(7)	C25−C26	1.408(4)
P6−C40	1.816(3)	C73−C74	1.408(4)
P6−C52	1.828(3)	C55−C56	1.385(4)
P6−C38	1.839(3)	C52−C57	1.408(4)
P6−Cu1	2.2697(7)	C45−C44	1.396(4)
P4−C67	1.832(3)	C76−C77	1.383(5)
P4−C65	1.835(3)	C76−C75	1.394(5)
P4−C79	1.822(3)	C84−C83	1.391(5)
P4−Cu2	2.2725(7)	C84−C79	1.387(4)
P3−C58	1.831(3)	C5−C6	1.385(5)
P3−C65	1.834(3)	C109−C110	1.400(4)
P3−C73	1.823(3)	C109−C108	1.398(5)
P3−Cu3	2.2648(7)	C82−C83	1.365(6)
P1−C4	1.831(3)	C82−C81	1.375(6)
P1−C19	1.821(3)	C114−Cu1	2.092(3)
P1−C11	1.830(3)	C114−Cu2	2.103(3)
P1−Cu1	2.2680(7)	C114−Cu3	2.384(3)
P5−C31	1.834(3)	C15−C14	1.395(5)
P5−C46	1.825(3)	C15−C16	1.366(5)
P5−C38	1.835(3)	C74−C75	1.383(4)
P5−Cu2	2.2728(8)	C51−C50	1.392(6)
F2−C94	1.367(4)	C57−C56	1.395(4)
F1−C106	1.368(4)	C26−C27	1.398(4)
C4−C9	1.389(4)	C41−C42	1.399(5)
C4−C5	1.399(4)	C7−C8	1.382(5)
C102−C101	1.211(4)	C7−C6	1.383(5)
C102−Cu1	2.172(3)	C110−C111	1.395(5)
C78−C73	1.400(4)	C47−C48	1.397(6)
C78−C77	1.391(4)	C20−C21	1.402(5)
C18−C17	1.393(5)	C34−C33	1.403(6)
C9−C8	1.390(5)	C23−C22	1.378(6)
C19−C24	1.396(4)	C99−C94	1.346(6)
C19−C20	1.379(5)	C43−C42	1.368(6)
C40−C45	1.397(4)	C21−C22	1.367(6)
C40−C41	1.385(4)	C108−C107	1.381(6)
C31−C36	1.401(4)	C106−C111	1.373(5)
C31−C32	1.383(4)	C106−C107	1.370(6)

Table S3. Selected bond angles (°) for **Cu$_3$-F**.

C13−P2−C11	98.06(13)	Cu1−C114−Cu3	70.26(9)
C13−P2−Cu3	122.67(9)	Cu2−C114−Cu3	74.23(9)
C25−P2−C13	105.42(13)	C16−C15−C14	121.2(3)
C25−P2−C11	105.14(13)	C15−C14−C13	119.3(3)
C25−P2−Cu3	113.54(10)	C75−C74−C73	120.8(3)
C11−P2−Cu3	109.79(10)	C74−C75−C76	119.9(3)
C40−P6−C52	103.55(13)	C50−C51−C46	119.1(5)
C40−P6−C38	105.13(13)	C56−C57−C52	119.9(3)
C40−P6−Cu1	118.78(10)	C27−C26−C25	119.7(3)
C52−P6−C38	101.75(13)	C58−C59−C60	120.4(3)
C52−P6−Cu1	115.75(10)	C40−C41−C42	120.4(3)
C38−P6−Cu1	110.08(9)	P1−C11−P2	110.24(15)
C67−P4−C65	104.79(13)	C8−C7−C6	119.6(3)
C67−P4−Cu2	120.58(9)	C55−C56−C57	120.5(3)
C65−P4−Cu2	111.59(9)	C111−C110−C109	120.8(3)
C79−P4−C67	100.60(13)	C84−C79−P4	117.6(2)
C79−P4−C65	103.81(13)	C84−C79−C80	117.9(3)
C79−P4−Cu2	113.65(9)	C80−C79−P4	124.2(2)
C58−P3−C65	104.51(12)	C71−C70−C69	120.4(3)
C58−P3−Cu3	117.76(9)	C16−C17−C18	119.7(3)
C65−P3−Cu3	111.05(9)	C30−C29−C28	119.8(3)
C73−P3−C58	101.70(12)	C70−C71−C72	120.8(3)
C73−P3−C65	104.34(13)	C7−C8−C9	120.5(3)
C73−P3−Cu3	115.96(9)	P5−C38−P6	108.89(15)
C4−P1−Cu1	120.85(10)	C34−C35−C36	122.1(4)
C19−P1−C4	103.97(13)	C35−C36−C31	119.8(3)
C19−P1−C11	104.14(13)	C67−C72−C71	119.5(3)
C19−P1−Cu1	112.47(9)	C27−C28−C29	120.1(3)
C11−P1−C4	102.42(13)	C43−C44−C45	119.8(3)
C11−P1−Cu1	111.30(9)	C97−C98−C99	120.0(3)
C31−P5−C38	102.45(13)	C7−C6−C5	120.6(3)
C31−P5−Cu2	116.02(9)	C19−C24−C23	119.9(3)
C46−P5−C31	103.49(14)	C28−C27−C26	120.5(3)
C46−P5−C38	102.36(14)	C31−C32−C33	120.6(3)
C46−P5−Cu2	119.63(12)	C94−C95−C96	117.4(3)
C38−P5−Cu2	110.75(9)	C46−C47−C48	118.9(5)
C9−C4−P1	122.4(2)	C19−C20−C21	119.3(3)
C9−C4−C5	119.4(3)	C35−C34−C33	118.5(3)
C5−C4−P1	118.3(2)	C22−C23−C24	120.1(3)
C101−C102−Cu1	135.6(2)	C94−C99−C98	119.0(3)

Table S4. Crystal data and structure refinement of **Cu_3-Cl**.

Identification code	Cu_3-Cl
Empirical formula	$C_{91}H_{74}Cl_2Cu_3F_6P_7$
Formula weight	1759.81
Temperature/K	100.00(10)
Crystal system	triclinic
Space group	P-1
a/Å	14.6965(2)
b/Å	17.7352(3)
c/Å	18.1319(2)
α/°	73.2110(10)
β/°	89.4850(10)
γ/°	88.3730(10)
Volume/Å^3	4522.73(11)
Z	2
ρ_{calc}g/cm^3	1.292
μ/mm^{-1}	2.975
F(000)	1800
Crystal size/mm^3	0.5 × 0.2 × 0.1
Radiation	Cu Kα (λ = 1.54184)
2θ range for data collection/°	5.206 to 154.626
Index ranges	$-18 \leq h \leq 18$, $-22 \leq k \leq 22$, $-21 \leq l \leq 22$
Reflections collected	56515
Independent reflections	18368 [R_{int} = 0.0830, R_{sigma} = 0.0678]
Data/restraints/parameters	18368/0/982
Goodness-of-fit on F^2	1.05
Final R indexes [I>=2σ (I)]	R_1 = 0.0654, wR_2 = 0.1960
Final R indexes [all data]	R_1 = 0.0739, wR_2 = 0.2139
Largest diff. peak/hole / e Å^{-3}	1.41/−0.94

Table S5. Selected bond lengths (Å) for **Cu$_3$-Cl**.

Cu1−Cu2	2.5138(7)	C19−C20	1.396(6)
Cu1−Cu3	2.6900(7)	C19−C24	1.397(5)
Cu1−P3	2.2683(9)	C1−C2	1.402(5)
Cu1−P2	2.2763(9)	C1−C6	1.395(5)
Cu1−C84	2.209(4)	C46−C47	1.394(6)
Cu1−C76	2.091(3)	C77−C76	1.218(5)
Cu2−Cu3	2.7020(7)	C7−C12	1.400(6)
Cu2−P1	2.2907(9)	C7−C8	1.396(6)
Cu2−P6	2.2737(9)	C13−C18	1.381(6)
Cu2−C84	2.231(4)	C13−C14	1.403(6)
Cu2−C76	2.118(4)	C32−C34	1.385(6)
Cu3−P5	2.2770(9)	C2−C3	1.392(6)
Cu3−P4	2.2868(10)	C6−C5	1.389(6)
Cu3−C84	2.069(3)	C57−C62	1.388(6)
Cu3−C76	2.398(4)	C57−C58	1.377(6)
Cl1−C89	1.734(4)	C27−C28	1.386(5)
P1−C44	1.829(4)	C12−C11	1.378(6)
P1−C50	1.834(3)	C68−C67	1.371(6)
P1−C38	1.820(4)	C28−C29	1.382(6)
P3−C63	1.824(4)	C4−C3	1.385(6)
P3−C69	1.824(4)	C4−C5	1.392(6)
P3−C75	1.838(3)	C38−C43	1.402(5)
P2−C33	1.816(4)	C38−C39	1.391(5)
P2−C26	1.825(3)	C83−C82	1.390(5)
P2−C50	1.840(4)	C37−C36	1.397(6)
P5−C19	1.821(4)	C8−C9	1.383(6)
P5−C13	1.832(3)	C18−C17	1.389(6)
P5−C25	1.846(4)	C43−C42	1.385(6)
P6−C1	1.816(4)	C62−C61	1.397(5)
P6−C7	1.830(4)	C20−C21	1.390(6)
P6−C25	1.843(3)	C70−C71	1.398(6)
P4−C51	1.818(4)	C65−C64	1.374(6)
P4−C57	1.836(4)	C65−C66	1.396(6)
P4−C75	1.841(4)	C30−C29	1.387(7)
Cl2−C81	1.755(4)	C30−C31	1.378(6)
P7−F3	1.595(3)	C39−C40	1.404(6)
P7−F6	1.586(3)	C82−C81	1.376(7)
P7−F5	1.598(3)	C79−C80	1.385(6)
P7−F1	1.604(3)	C52−C53	1.385(7)
P7−F4	1.599(3)	C36−C35	1.377(7)

Table S6. Selected bond angles (°) for **Cu3-Cl**.

Cu2−Cu1−Cu3	62.454(18)	Cu1−C84−Cu2	68.97(11)
P3−Cu1−Cu2	152.52(3)	Cu3−C84−Cu1	77.84(12)
P3−Cu1−Cu3	95.34(3)	Cu3−C84−Cu2	77.75(12)
P3−Cu1−P2	109.87(3)	C85−C84−Cu1	132.9(3)
P2−Cu1−Cu2	96.38(3)	C85−C84−Cu2	134.2(3)
P2−Cu1−Cu3	150.23(3)	C85−C84−Cu3	138.2(3)
C84−Cu1−Cu2	55.93(9)	C45−C44−P1	118.6(3)
C84−Cu1−Cu3	48.77(8)	C49−C44−P1	123.1(3)
C84−Cu1−P3	122.54(10)	C49−C44−C45	118.1(3)
C84−Cu1−P2	102.63(9)	C91−C90−C89	119.3(3)
C76−Cu1−Cu2	53.83(10)	C90−C89−Cl1	120.0(3)
C76−Cu1−Cu3	58.61(10)	C90−C89−C88	120.9(3)
C76−Cu1−P3	101.71(10)	C88−C89−Cl1	119.1(3)
C76−Cu1−P2	127.27(11)	C70−C69−P3	119.7(3)
C76−Cu1−C84	93.97(13)	C70−C69−C74	119.0(4)
Cu1−Cu2−Cu3	61.969(18)	C74−C69−P3	121.1(3)
P1−Cu2−Cu1	95.90(3)	C52−C51−P4	119.4(3)
P1−Cu2−Cu3	149.39(3)	C52−C51−C56	119.0(4)
P6−Cu2−Cu1	150.42(3)	C56−C51−P4	121.5(3)
P6−Cu2−Cu3	95.20(3)	C20−C19−P5	119.5(3)
P6−Cu2−P1	111.69(4)	C20−C19−C24	118.8(4)
C84−Cu2−Cu1	55.10(9)	C24−C19−P5	121.7(3)
C84−Cu2−Cu3	48.46(8)	C2−C1−P6	119.9(3)
C84−Cu2−P1	102.14(9)	C6−C1−P6	121.5(3)
C84−Cu2−P6	124.66(9)	C6−C1−C2	118.5(3)
C76−Cu2−Cu1	52.82(9)	P1−C50−P2	109.59(17)
C76−Cu2−Cu3	58.16(10)	C87−C88−C89	119.1(3)
C76−Cu2−P1	126.74(10)	C45−C46−C47	119.6(4)
C76−Cu2−P6	100.01(9)	C76−C77−C78	177.5(4)
C76−Cu2−C84	92.59(14)	C12−C7−P6	119.6(3)
Cu1−Cu3−Cu2	55.578(17)	C8−C7−P6	122.1(3)
P5−Cu3−Cu1	146.44(3)	C8−C7−C12	117.8(4)
P5−Cu3−Cu2	91.76(3)	C18−C13−P5	123.2(3)
P5−Cu3−P4	118.48(4)	C18−C13−C14	119.1(4)
P5−Cu3−C76	106.16(8)	C14−C13−P5	117.7(3)
P4−Cu3−Cu1	92.26(3)	C34−C32−C33	120.0(4)
P4−Cu3−Cu2	147.07(3)	C3−C2−C1	120.9(3)
P4−Cu3−C76	106.22(9)	C5−C6−C1	120.7(3)
C84−Cu3−Cu1	53.39(10)	C62−C57−P4	121.4(3)
C84−Cu3−Cu2	53.79(10)	C58−C57−P4	120.5(3)

Table S7. Crystal data and structure refinement of **Cu_3-Br**.

Identification code	Cu_3-Br
Empirical formula	$C_{91}H_{74}Br_2Cu_3F_6P_7$
Formula weight	1848.73
Temperature/K	100.01(10)
Crystal system	triclinic
Space group	P-1
a/Å	14.7445(2)
b/Å	17.7915(3)
c/Å	18.0501(3)
α/°	73.2970(10)
β/°	89.5660(10)
γ/°	88.6640(10)
Volume/Å^3	4534.01(13)
Z	2
ρ_{calc}g/cm^3	1.354
μ/mm^{-1}	3.458
F(000)	1872
Crystal size/mm^3	0.304 × 0.208 × 0.186
Radiation	Cu Kα (λ = 1.54184)
2θ range for data collection/°	5.112 to 152.26
Index ranges	$-18 \leq h \leq 18$, $-22 \leq k \leq 21$, $-22 \leq l \leq 22$
Reflections collected	58222
Independent reflections	18369 [R_{int} = 0.0318, R_{sigma} = 0.0248]
Data/restraints/parameters	18369/0/982
Goodness-of-fit on F^2	1.05
Final R indexes [I>=2σ (I)]	R_1 = 0.0784, wR_2 = 0.2351
Final R indexes [all data]	R_1 = 0.0792, wR_2 = 0.2356
Largest diff. peak/hole / e Å^{-3}	2.33/−1.52

Table S8. Selected bond lengths (Å) for **Cu_3-Br**.

Br1−C6	1.893(5)	C86−C91	1.383(8)
Br2−C89	1.896(6)	C10−C9	1.400(7)
Cu1−Cu2	2.7133(10)	C10−C11	1.382(7)
Cu1−Cu3	2.7023(10)	C15−C20	1.397(8)
Cu1−P1	2.2819(14)	C29−C30	1.390(8)
Cu1−P6	2.2831(14)	C9−C14	1.375(8)
Cu1−C84	2.456(5)	C22−C23	1.403(7)
Cu1−C1	2.095(5)	C22−C27	1.375(8)
Cu2−Cu3	2.5211(10)	C41−C40	1.383(8)
Cu2−P2	2.2726(14)	C41−C42	1.403(8)
Cu2−P3	2.2916(14)	C35−C36	1.391(8)
Cu2−C84	2.174(4)	C5−C4	1.394(7)
Cu2−C1	2.244(5)	C52−C51	1.382(8)
Cu3−P5	2.2649(14)	C83−C78	1.387(8)
Cu3−P4	2.2753(14)	C83−C82	1.374(8)
Cu3−C84	2.143(4)	C45−C40	1.399(7)
Cu3−C1	2.222(5)	C45−C44	1.388(8)
P2−C72	1.820(5)	C7−C8	1.389(8)
P2−C71	1.835(5)	C11−C12	1.364(8)
P2−C78	1.833(5)	C53−C54	1.382(8)
P3−C21	1.844(5)	C53−C58	1.382(8)
P3−C15	1.826(5)	C30−C31	1.388(9)
P3−C9	1.829(5)	C78−C79	1.390(7)
P1−C59	1.830(5)	C33−C32	1.420(8)
P1−C71	1.846(5)	C77−C76	1.386(8)
P1−C65	1.825(5)	C65−C66	1.392(8)
P6−C47	1.822(5)	C65−C70	1.392(7)
P6−C46	1.843(5)	C23−C24	1.386(8)
P6−C53	1.839(5)	C87−C88	1.387(8)
P5−C34	1.826(5)	C48−C49	1.399(8)
P5−C46	1.834(5)	C44−C43	1.401(8)
P5−C40	1.827(5)	C64−C63	1.399(8)
P4−C28	1.813(5)	C66−C67	1.386(8)
P4−C21	1.831(5)	C31−C32	1.371(9)
P4−C22	1.838(5)	C39−C38	1.377(8)
P7−F2	1.616(4)	C24−C25	1.377(9)
P7−F6	1.612(4)	C20−C19	1.391(8)
P7−F1	1.598(4)	C17−C18	1.369(10)
P7−F3	1.558(5)	C54−C55	1.382(8)
P7−F4	1.600(5)	C80−C79	1.406(8)

Table S9. Selected bond angles (°) for **Cu_3-Br**.

Cu3−Cu1−Cu2	55.49(2)	C35−C34−P5	119.0(4)
P1−Cu1−Cu2	91.52(4)	C35−C34−C39	118.8(5)
P1−Cu1−Cu3	146.06(5)	C39−C34−P5	122.0(4)
P1−Cu1−P6	118.75(5)	C64−C59−P1	122.8(4)
P1−Cu1−C84	105.38(10)	C64−C59−C60	118.9(5)
P6−Cu1−Cu2	146.84(5)	C60−C59−P1	118.3(4)
P6−Cu1−Cu3	92.17(4)	C29−C28−P4	120.6(4)
P6−Cu1−C84	105.42(10)	C33−C28−P4	119.9(4)
C84−Cu1−Cu2	49.44(10)	C33−C28−C29	119.5(5)
C84−Cu1−Cu3	48.81(10)	P4−C21−P3	109.9(3)
C1−Cu1−Cu2	53.80(13)	C8−C3−C2	121.2(5)
C1−Cu1−Cu3	53.38(13)	C8−C3−C4	118.4(5)
C1−Cu1−P1	116.13(13)	C4−C3−C2	120.4(4)
C1−Cu1−P6	115.30(13)	C84−C85−C86	175.9(6)
C1−Cu1−C84	90.19(17)	P5−C46−P6	110.2(3)
Cu3−Cu2−Cu1	62.03(3)	C88−C89−Br2	120.4(5)
P2−Cu2−Cu1	95.15(4)	C88−C89−C90	120.7(5)
P2−Cu2−Cu3	150.49(5)	C90−C89−Br2	118.8(4)
P2−Cu2−P3	111.57(5)	C73−C72−P2	119.2(4)
P3−Cu2−Cu1	149.55(5)	C73−C72−C77	119.3(5)
P3−Cu2−Cu3	95.97(4)	C77−C72−P2	121.5(4)
C84−Cu2−Cu1	59.11(12)	C15−C16−C17	119.5(6)
C84−Cu2−Cu3	53.70(11)	C72−C73−C74	119.6(5)
C84−Cu2−P2	99.43(12)	C87−C86−C85	120.8(5)
C84−Cu2−P3	126.38(12)	C91−C86−C85	122.2(5)
C84−Cu2−C1	94.06(17)	C91−C86−C87	117.0(5)
C1−Cu2−Cu1	48.88(13)	C11−C10−C9	121.5(5)
C1−Cu2−Cu3	55.21(12)	C16−C15−P3	119.5(4)
C1−Cu2−P2	124.73(13)	C16−C15−C20	119.3(5)
C1−Cu2−P3	101.88(13)	C20−C15−P3	121.2(4)
Cu2−Cu3−Cu1	62.48(3)	C30−C29−C28	121.0(5)
P5−Cu3−Cu1	95.21(4)	C10−C9−P3	118.4(4)
P5−Cu3−Cu2	152.54(5)	C14−C9−P3	124.0(4)
P5−Cu3−P4	110.01(5)	C14−C9−C10	117.3(5)
P4−Cu3−Cu1	150.16(5)	C23−C22−P4	119.6(4)
P4−Cu3−Cu2	96.28(4)	C27−C22−P4	121.0(4)
C84−Cu3−Cu1	59.59(12)	C27−C22−C23	119.1(5)
C84−Cu3−Cu2	54.85(12)	C40−C41−C42	120.3(5)
C84−Cu3−P5	101.01(12)	C34−C35−C36	120.2(5)
C84−Cu3−P4	126.92(12)	C4−C5−C6	118.1(5)

Table S10. Crystal data and structure refinement of **Cu_3-I**.

Identification code	Cu_3-I
Empirical formula	$C_{91}H_{74}Cu_3F_6I_2P_7$
Formula weight	1942.71
Temperature/K	100.01(10)
Crystal system	monoclinic
Space group	$P2_1/n$
a/Å	14.67800(10)
b/Å	24.4754(3)
c/Å	23.6388(2)
α/°	90
β/°	93.5100(10)
γ/°	90
Volume/$Å^3$	8476.31(14)
Z	4
$\rho_{calc}g/cm^3$	1.522
μ/mm^{-1}	8.314
F(000)	3888
Crystal size/mm^3	0.355 × 0.348 × 0.184
Radiation	Cu Kα (λ = 1.54184)
2θ range for data collection/°	5.202 to 152.296
Index ranges	$-18 \leq h \leq 16, -30 \leq k \leq 30, -29 \leq l \leq 29$
Reflections collected	59860
Independent reflections	17150 [R_{int} = 0.0462, R_{sigma} = 0.0337]
Data/restraints/parameters	17150/0/983
Goodness-of-fit on F^2	1.056
Final *R* indexes [I>=2σ (I)]	R_1 = 0.0509, wR_2 = 0.1422
Final *R* indexes [all data]	R_1 = 0.0515, wR_2 = 0.1430
Largest diff. peak/hole / e $Å^{-3}$	3.29/−2.11

Table S11. Selected bond lengths (Å) for **Cu_3-I**.

I2−C14	2.095(4)	C72−C71	1.396(5)
I1−C6	2.094(4)	C86−C87	1.387(5)
Cu1−Cu2	2.6522(6)	C86−C91	1.397(5)
Cu1−Cu3	2.5693(7)	C62−C63	1.385(5)
Cu1−P6	2.2819(9)	C60−C59	1.396(5)
Cu1−P1	2.2710(9)	C81−C82	1.394(5)
Cu1−C1	2.157(3)	C43−C44	1.391(5)
Cu1−C9	2.235(4)	C66−C65	1.386(5)
Cu2−Cu3	2.6469(7)	C7−C6	1.385(6)
Cu2−P3	2.2908(9)	C7−C8	1.391(6)
Cu2−P2	2.2849(9)	C73−C74	1.400(5)
Cu2−C1	2.250(4)	C73−C78	1.388(5)
Cu2−C9	2.289(3)	C14−C13	1.382(6)
Cu3−P5	2.3003(9)	C14−C15	1.391(6)
Cu3−P4	2.2826(9)	C6−C5	1.381(6)
Cu3−C1	2.304(3)	C3−C4	1.399(5)
Cu3−C9	2.191(4)	C3−C2	1.443(5)
P3−C17	1.831(4)	C3−C8	1.409(5)
P3−C23	1.824(4)	C47−C46	1.393(5)
P3−C29	1.838(3)	C11−C12	1.393(6)
P6−C55	1.831(3)	C11−C16	1.396(6)
P6−C54	1.841(3)	C11−C10	1.489(6)
P6−C61	1.815(4)	C25−C24	1.380(5)
P1−C67	1.820(3)	C25−C26	1.389(6)
P1−C79	1.833(3)	C87−C88	1.386(5)
P1−C73	1.818(3)	C36−C37	1.398(5)
P2−C80	1.830(4)	C36−C41	1.402(5)
P2−C79	1.830(3)	C45−C44	1.378(5)
P2−C86	1.827(4)	C45−C46	1.397(6)
P5−C54	1.842(3)	C4−C5	1.399(5)
P5−C48	1.824(3)	C53−C52	1.389(6)
P5−C42	1.831(4)	C71−C70	1.386(6)
P4−C30	1.821(4)	C13−C12	1.399(6)
P4−C29	1.843(3)	C22−C21	1.390(6)
P4−C36	1.828(4)	C35−C34	1.382(6)
P7−F7	1.598(2)	C37−C38	1.394(6)
P7−F3	1.597(3)	C91−C90	1.379(5)
P7−F1	1.589(3)	C33−C32	1.390(6)
P7−F2	1.588(3)	C33−C34	1.394(6)
P7−F4	1.585(3)	C59−C58	1.388(6)

Table S12. Selected bond angles (°) for **Cu_3-I**.

Cu3−Cu1−Cu2	60.893(18)	C55−C56−C57	120.1(3)
P6−Cu1−Cu2	154.07(3)	C24−C23−P3	120.2(3)
P6−Cu1−Cu3	93.63(3)	C28−C23−P3	120.6(3)
P1−Cu1−Cu2	95.37(3)	C28−C23−C24	119.3(3)
P1−Cu1−Cu3	152.71(3)	C70−C69−C68	120.0(4)
P1−Cu1−P6	110.52(3)	C50−C49−C48	120.7(4)
C1−Cu1−Cu2	54.61(9)	C35−C30−P4	119.5(3)
C1−Cu1−Cu3	57.57(9)	C35−C30−C31	119.1(3)
C1−Cu1−P6	109.21(9)	C31−C30−P4	121.4(3)
C1−Cu1−P1	121.46(9)	C43−C42−P5	117.9(3)
C1−Cu1−C9	97.20(13)	C43−C42−C47	118.8(3)
C9−Cu1−Cu2	55.06(8)	C47−C42−P5	123.1(3)
C9−Cu1−Cu3	53.73(9)	C72−C67−P1	122.5(3)
C9−Cu1−P6	115.25(9)	C72−C67−C68	119.0(3)
C9−Cu1−P1	102.64(9)	C68−C67−P1	118.5(3)
Cu3−Cu2−Cu1	58.006(17)	C62−C61−P6	123.6(3)
P3−Cu2−Cu1	150.86(3)	C66−C61−P6	117.9(3)
P3−Cu2−Cu3	92.98(3)	C66−C61−C62	118.5(3)
P2−Cu2−Cu1	93.02(3)	Cu1−C9−Cu2	71.78(11)
P2−Cu2−Cu3	150.27(3)	Cu3−C9−Cu1	70.96(12)
P2−Cu2−P3	115.34(3)	Cu3−C9−Cu2	72.39(11)
P2−Cu2−C9	106.83(9)	C10−C9−Cu1	128.4(3)
C1−Cu2−Cu1	51.42(8)	C10−C9−Cu2	137.5(3)
C1−Cu2−Cu3	55.43(8)	C10−C9−Cu3	144.8(3)
C1−Cu2−P3	116.47(8)	C71−C72−C67	120.2(3)
C1−Cu2−P2	113.56(9)	P2−C79−P1	109.90(17)
C1−Cu2−C9	93.10(13)	C87−C86−P2	118.7(3)
C9−Cu2−Cu1	53.17(9)	C87−C86−C91	119.5(3)
C9−Cu2−Cu3	52.10(9)	C91−C86−P2	121.7(3)
C9−Cu2−P3	108.55(9)	C63−C62−C61	120.7(4)
Cu1−Cu3−Cu2	61.101(18)	P3−C29−P4	109.14(17)
P5−Cu3−Cu1	96.30(3)	C55−C60−C59	120.4(3)
P5−Cu3−Cu2	153.13(3)	C80−C81−C82	120.6(4)
P5−Cu3−C1	101.89(9)	C44−C43−C42	120.7(3)
P4−Cu3−Cu1	151.91(3)	C65−C66−C61	120.8(3)
P4−Cu3−Cu2	94.83(3)	C69−C68−C67	120.4(3)
P4−Cu3−P5	110.30(4)	C6−C7−C8	119.6(4)
P4−Cu3−C1	126.49(8)	C74−C73−P1	117.0(3)
C1−Cu3−Cu1	52.20(8)	C78−C73−P1	123.8(3)
C1−Cu3−Cu2	53.51(9)	C78−C73−C74	118.6(3)

Table S13. Photophysical properties of copper(I) cluster complexes reported in the literature and this work.

**Clusters**	τ_{298K} / μs	λ_{em} / nm	**Ref.**
$[Cu_3(dppm)_3(C{\equiv}C\text{-}C{\equiv}CH\text{-}{}^nC_6H_{13})_2]PF_6$	15.2	616	ref [1]
$[Cu_3(dppm)_3(C{\equiv}C^tBu)Cl]PF_6$	33	535	ref [2]
$[Cu_3(dppm)_3(C{\equiv}CC_6H_4OMe\text{-}p)(C{\equiv}CC_6H_4OEt\text{-}p)]PF_6$	17.6	475, 502	ref [3]
$[Cu_3(dppm)_3(C{\equiv}CC_6H_2\text{-}2,5\text{-}Me_2\text{-}C{\equiv}C\text{-}Re(bpy)(CO)_3)_2]PF_6$	0.13	618	ref [4]
$[Cu_3(dppm)_3(C{\equiv}CC(O)NEt_2)_2](ClO_4)$	15.4	443	ref [5]
$[Cu_3(dppm)_3(C{\equiv}CC_6H_4\text{-}4\text{-}NHC(O)NHC_6H_4\text{-}4\text{-}CF_3)_2F]_\infty$	54.1	488, 531	ref [6]
$[Cu_3(dppm)_3(C{\equiv}CC_6H_4OMe\text{-}p)_2]$ PF_6	63.8	450, 482	ref [7]
$[Cu_9(PhSe)_6(PPh_2O_2)_3]$	16.4	648, 684	ref [8]
$[Cu_9(CH_3OPhS)_6(PPh_2O_2)_3]$	18.6	648, 680	ref [8]
$Cu_4I_4L_4$	6.6	425, 605	ref [9]
$[Cu_4I_4(4\text{-dpda})_4]$	2.54	540	ref [10]
$[Cu_4I_4[PPh_2(C_6H_4\text{-}CH_2OH)]_4]$	3.91	542	ref [11]
**Cu_3-F**	30.64	491	This work
**Cu_3-Cl**	72.19	502	This work
**Cu_3-Br**	114.56	511	This work
**Cu_3-I**	18.08	564	This work

Table S14. Calculated Triplet Excitation Energies ($\Delta E(S_0 \rightarrow T_1)$), Spin-Orbit Coupling (SOC) Constants between S_1 and T_1, Calculated Emission Wavelengths (Cal. λ_{em}), and Experimental Emission Wavelengths (Expt. λ_{em}) for Cu_3-X Clusters.

Clusters	$\Delta E(S_0 - T_1)$ (eV)	SOC (cm^{-1})	Cal. λ_{em} (nm) [a]	Expt. λ_{em} (nm)
**Cu_3-F**	2.79	1.59	491	491
**Cu_3-Cl**	2.73	2.83	501	502
**Cu_3-Br**	2.72	6.76	502	511
**Cu_3-I**	2.66	14.6	512	564

[a] the calculated emission wavelengths are consistently red shifted by 47 nm.

Table S15. Molecular orbitals contributing to the T_1 excited state (responsible for $T_1 \rightarrow S_0$ phosphorescence) of Cu_3-X Clusters.

Clusters	$\Delta E(S_0 - T_1)$ (eV)	Transition Molecular Orbital
**Cu_3-F**	2.79	HOMO → LUMO+1 **18.3%**
		HOMO → LUMO+2 **10.7%**
		HOMO-1 → LUMO+1 **16.5%**
		HOMO-1 →LUMO+2 **13.0%**
**Cu_3-Cl**	2.73	HOMO-1 → LUMO+1 **23.6%**
		HOMO-1 → LUMO+2 **12.5%**
		HOMO-2 → LUMO+2 **8.62%**
**Cu_3-Br**	2.72	HOMO-1 → LUMO+1 **25.3%**
		HOMO-1 → LUMO+2 **17.1%**
		HOMO-2 → LUMO+1 **10.4%**
**Cu_3-I**	2.66	HOMO → LUMO **29.0%**
		HOMO-1 → LUMO **30.0%**

Reference

(1) Lo, W.-Y.; Lam, C.-H.; Yam, V. W.-W.; Zhu, N.; Cheung, K.-K.; Fathallah, S.; Messaoudi, S.; Le Guennic, B.; Kahlal, S.; Halet, J.-F. Synthesis, Photophysics, Electrochemistry, Theoretical, and Transient Absorption Studies of Luminescent Copper(I) and Silver(I) Diynyl Complexes. X-ray Crystal Structures of $[Cu_3(\mu\text{-dppm})_3(\mu_3\text{-}\eta^1\text{-C≡CC≡CPh})_2]PF_6$ and $[Cu_3(\mu\text{-dppm})_3(\mu_3\text{-}\eta^1\text{-C≡CC≡CH})_2]PF_6$. *J. Am. Chem. Soc.* **2004**, *126*, 7300–7310.

(2) Yam, V. W. W.; Lee, W. K.; Lai, T. F. Synthesis, spectroscopy, and electrochemistry of trinuclear copper(I) acetylides. X-ray crystal structure of $[Cu_3(\mu\text{-}Ph_2PCH_2PPh_2)_3(\mu_3\text{-}\eta^1\text{-C≡C}^t\text{Bu})(\mu_3\text{-Cl})]PF_6$. *Organometallics* **1993**, *12*, 2383–2387.

(3) Yam, V. W.-W.; Fung, W. K.-M.; Cheung, K.-K. Synthesis, Luminescence, and Electrochemistry of Mix-Capped Trinuclear Copper(I) Acetylide Complexes. X-ray Crystal Structures of $[Cu_3(\mu\text{-dppm})_3(\mu_3\text{-}\eta^1\text{-C≡CC}_6H_4OMe\text{-}p)(\mu_3\text{-}\eta^1\text{-C≡CC}_6H_4OEt\text{-}p)]PF_6$ and $[Cu_3(\mu\text{-dppm})_3(\mu_3\text{-}\eta^1\text{-C≡CC}_6H_4OMe\text{-}p)(\mu_2\text{-}\eta^1\text{-C≡CC}_6H_4NO_2\text{-}p)]PF_6$. *Organometallics* **1998**, *17*, 3293–3298.

(4) Yam, V. W.-W.; Lo, W.-Y.; Lam, C.-H.; Fung, W. K.-M.; Wong, K. M.-C.; Lau, V. C.-Y.; Zhu, N. Synthesis and luminescence behaviour of mixed-metal rhenium(I)–copper(I) and –silver(I) alkynyl complexes. *Coord. Chem. Rev.* **2003**, *245*, 39–47.

(5) Zhang, M.; Su, B.-C.; Li, C.-L.; Shen, Y.; Lam, C.-K.; Feng, X.-L.; Chao, H.-Y. Trinuclear copper(I) acetylide complexes bearing carbonyl moiety: Synthesis, characterization, and photophysical properties. *J. Organomet. Chem.* **2011**, *696*, 2654–2659.

(6) Zhou, Y.-P.; Wei, Z.-W.; Lin, Z.-J.; Ling, H.-T.; Guo, Z.; Zhang, M.; Lam, C.-K.; Ye, B.-H.; Chao, H.-Y. Diverse binding of important anions in 1-D tricopper anion coordination polymer (ACP) architectures. *CrystEngComm* **2017**, *19*, 2349–2358.

(7) Yam, V. W.-W.; Fung, W. K.-M.; Cheung, K.-K. Luminescence Behavior of Polynuclear Alkynylcopper(I) Phosphines. *J. Cluster Sci.* **1999**, *10*, 37–69.

(8) Du, P.; Sachurilatu; Jiang, W.; Wei, J.; Li, S.; Shen, H. Ligand effects in photoluminescence of copper nanoclusters. *Dalton Trans.* **2024**, *53*, 15190–15197.

(9) Egly, J.; Bissessar, D.; Achard, T.; Heinrich, B.; Steffanut, P.; Mauro, M.; Bellemin-Laponnaz, S. Copper(I) complexes with remotely functionalized phosphine ligands: Synthesis, structural variety, photophysics and effect onto the optical properties. *Inorg. Chim. Acta* **2021**, *514*, 119971.

(10) Shan, X.-C.; Jiang, F.-L.; Chen, L.; Wu, M.-Y.; Pan, J.; Wan, X.-Y.; Hong, M.-C. Using cuprophilicity as a multi-responsive chromophore switching color in response to temperature, mechanical force and solvent vapors. *J. Mater. Chem. C* **2013**, *1*, 4339–4349.

(11) Utrera-Melero, R.; Huitorel, B.; Cordier, M.; Mevellec, J.-Y.; Massuyeau, F.; Latouche, C.; Martineau-Corcos, C.; Perruchas, S. Combining Theory and Experiment to Get Insight into the Amorphous Phase of Luminescent Mechanochromic Copper Iodide Clusters. *Inorg. Chem.* **2020**, *59*, 13607–13620.
